# Blood pressure-lowering treatment for prevention of major cardiovascular diseases in people with and without type 2 diabetes: an individual participant-level data meta-analysis

**DOI:** 10.1016/S2213-8587(22)00172-3

**Published:** 2022-09

**Authors:** Milad Nazarzadeh, Zeinab Bidel, Dexter Canoy, Emma Copland, Derrick A Bennett, Abbas Dehghan, George Davey Smith, Rury R Holman, Mark Woodward, Ajay Gupta, Amanda I Adler, Malgorzata Wamil, Naveed Sattar, William C Cushman, Richard J McManus, Koon Teo, Barry R Davis, John Chalmers, Carl J Pepine, Kazem Rahimi, Blood Pressure Lowering Treatment Trialists' Collaboration, L Agodoa, A Algra, F W Asselbergs, N Beckett, E Berge, H Black, F P J Brouwers, M Brown, C J Bulpitt, B Byington, J Cutler, R B Devereaux, J Dwyer, R Estacio, R Fagard, K Fox, T Fukui, Y Imai, M Ishii, S Julius, Y Kanno, S E Kjeldsen, J Kostis, K Kuramoto, J Lanke, E Lewis, J Lewis, M Lievre, L H Lindholm, S Lueders, S MacMahon, G Mancia, M Matsuzaki, M H Mehlum, S Nissen, H Ogawa, T Ogihara, T Ohkubo, C Palmer, A Patel, M Pfeffer, N R Poulter, H Rakugi, G Reboldi, C Reid, G Remuzzi, P Ruggenenti, T Saruta, J Schrader, R Schrier, P Sever, P Sleight, J A Staessen, H Suzuki, L Thijs, K Ueshima, S Umemoto, W H van Gilst, P Verdecchia, K Wachtell, P Whelton, L Wing, Y Yui, S Yusuf, A Zanchetti, Z Y Zhang, C Anderson, C Baigent, BM Brenner, R Collins, D de Zeeuw, J Lubsen, E Malacco, B Neal, V Perkovic, B Pitt, A Rodgers, P Rothwell, G Salimi-Khorshidi, J Sundström, F Turnbull, G Viberti, J Wang

**Affiliations:** a Deep Medicine, Oxford Martin School, University of Oxford, Oxford, UK; b Nuffield Department of Women's and Reproductive Health, Medical Science Division, University of Oxford, Oxford, UK; c NIHR Oxford Biomedical Research Centre, Oxford University Hospitals NHS Foundation Trust, Oxford, UK; d Clinical Trial Service Unit and Epidemiological Studies Unit, Nuffield Department of Population Health, University of Oxford, Oxford, UK; e Department of Epidemiology and Biostatistics, School of Public Health, Imperial College London, London, UK; f MRC-PHE Centre for Environment and Health, School of Public Health, Imperial College London, Norfolk Place, London, UK; g MRC Integrative Epidemiology Unit, University of Bristol, Bristol, UK; h Diabetes Trials Unit, Oxford Centre for Diabetes, Endocrinology and Metabolism, Radcliffe Department of Medicine, University of Oxford, Oxford, UK; i The George Institute for Global Health, School of Public Health, Imperial College London, London, UK; j The George Institute for Global Health, University of New South Wales, Sydney, NSW, Australia; k William Harvey Research Institute, Queen Mary University of London, London, UK; l Institute of Cardiovascular and Medical Sciences, University of Glasgow, Glasgow, UK; m Department of Preventive Medicine, University of Tennessee Health Science Center, Memphis, TN, USA; n Nuffield Department of Primary Care Health Sciences, University of Oxford, Oxford, UK; o Population Health Research Institute, McMaster University, Hamilton, ON, Canada; p University of Texas School of Public Health, Houston, TX, USA; q Department of Medicine, University of Florida, Gainesville, FL, USA

## Abstract

**Background:**

Controversy exists as to whether the threshold for blood pressure-lowering treatment should differ between people with and without type 2 diabetes. We aimed to investigate the effects of blood pressure-lowering treatment on the risk of major cardiovascular events by type 2 diabetes status, as well as by baseline levels of systolic blood pressure.

**Methods:**

We conducted a one-stage individual participant-level data meta-analysis of major randomised controlled trials using the Blood Pressure Lowering Treatment Trialists' Collaboration dataset. Trials with information on type 2 diabetes status at baseline were eligible if they compared blood pressure-lowering medications versus placebo or other classes of blood pressure-lowering medications, or an intensive versus a standard blood pressure-lowering strategy, and reported at least 1000 persons-years of follow-up in each group. Trials exclusively on participants with heart failure or with short-term therapies and acute myocardial infarction or other acute settings were excluded. We expressed treatment effect per 5 mm Hg reduction in systolic blood pressure on the risk of developing a major cardiovascular event as the primary outcome, defined as the first occurrence of fatal or non-fatal stroke or cerebrovascular disease, fatal or non-fatal ischaemic heart disease, or heart failure causing death or requiring hospitalisation. Cox proportional hazard models, stratified by trial, were used to estimate hazard ratios (HRs) separately by type 2 diabetes status at baseline, with further stratification by baseline categories of systolic blood pressure (in 10 mm Hg increments from <120 mm Hg to ≥170 mm Hg). To estimate absolute risk reductions, we used a Poisson regression model over the follow-up duration. The effect of each of the five major blood pressure-lowering drug classes, including angiotensin-converting enzyme inhibitors, angiotensin II receptor blockers, β blockers, calcium channel blockers, and thiazide diuretics, was estimated using a network meta-analysis framework. This study is registered with PROSPERO, CRD42018099283.

**Findings:**

We included data from 51 randomised clinical trials published between 1981 and 2014 involving 358 533 participants (58% men), among whom 103 325 (29%) had known type 2 diabetes at baseline. The baseline mean systolic/diastolic blood pressure of those with and without type 2 diabetes was 149/84 mm Hg (SD 19/11) and 153/88 mm Hg (SD 21/12), respectively. Over 4·2 years median follow-up (IQR 3·0–5·0), a 5 mm Hg reduction in systolic blood pressure decreased the risk of major cardiovascular events in both groups, but with a weaker relative treatment effect in participants with type 2 diabetes (HR 0·94 [95% CI 0·91–0·98]) compared with those without type 2 diabetes (0·89 [0·87–0·92]; p_interaction_=0·0013). However, absolute risk reductions did not differ substantially between people with and without type 2 diabetes because of the higher absolute cardiovascular risk among participants with type 2 diabetes. We found no reliable evidence for heterogeneity of treatment effects by baseline systolic blood pressure in either group. In keeping with the primary findings, analysis using stratified network meta-analysis showed no evidence that relative treatment effects differed substantially between participants with type 2 diabetes and those without for any of the drug classes investigated.

**Interpretation:**

Although the relative beneficial effects of blood pressure reduction on major cardiovascular events were weaker in participants with type 2 diabetes than in those without, absolute effects were similar. The difference in relative risk reduction was not related to the baseline blood pressure or allocation to different drug classes. Therefore, the adoption of differential blood pressure thresholds, intensities of blood pressure lowering, or drug classes used in people with and without type 2 diabetes is not warranted.

**Funding:**

British Heart Foundation, UK National Institute for Health Research, and Oxford Martin School.

**Research in context**

**Evidence before this study**

We searched PubMed, Web of Science, and Scopus for studies published between Jan 1, 1966, and Dec 1, 2021, using search terms “hypertension,” “blood pressure”, and “diabetes”, with no language restrictions. Several individual trials of blood pressure-lowering treatment in people with or without diabetes were identified, but their findings were conflicting. We found meta-analyses of trials that included only participants with type 2 diabetes. Two conventional meta-analyses found that blood pressure-lowering treatment reduced the risk of major cardiovascular events in this population; however, when trials were stratified by baseline systolic blood pressure, notable heterogeneity was observed; one study showed no effect in people with a baseline systolic blood pressure less of than 140 mm Hg, whereas another reported a harmful effect. Additionally, an individual-level participant data meta-analysis from the previous cycle of the Blood Pressure Lowering Treatment Trialists' Collaboration indicated that antihypertensive treatment reduced the risk of major cardiovascular events to nearly the same extent in people with and without type 2 diabetes. The study, however, had limited statistical power, analyses were not standardised for varying magnitudes of blood pressure-lowering, and stratification by baseline blood pressure levels was not performed.

**Added value of this study**

Blood pressure-lowering treatment reduced the risk of major cardiovascular events in people with and without type 2 diabetes in our individual participant-level data meta-analysis of major pharmacological blood pressure-lowering trials involving 103 325 participants with type 2 diabetes and 255 208 participants without type 2 diabetes. However, the relative effects were weaker in people with established type 2 diabetes than in those without. Nonetheless, because participants with type 2 diabetes were at a higher risk of major cardiovascular events, the absolute risk reductions between the two groups did not differ. Investigation of the underlying reasons for the heterogeneous relative effects suggested that the differences were not substantially influenced by the levels of systolic blood pressure at baseline or types of antihypertensive drugs used.

**Implications of all the available evidence**

Our analyses challenge the adoption of differential blood pressure thresholds, intensities or drug classes in people with and without type 2 diabetes. This study calls for the removal of specific blood pressure thresholds when selecting people with type 2 diabetes for antihypertensive therapy.

## Introduction

Diabetes is a major cause of death, cardiovascular complications, and health-care burden worldwide.^1^ People with type 2 diabetes who have high blood pressure are at an increased risk of morbidity and death from major cardiovascular events.^2^ However, there are inadequate randomised controlled trial data to determine if the benefit of blood pressure-lowering treatment differs in people with type 2 diabetes versus those without this metabolic condition. Similarly, there is uncertainty around initiating blood pressure reduction therapy at a specific blood pressure threshold, particularly in people with normal or high-to-normal blood pressure levels.

These uncertainties stem mostly from the disparate findings of the Action to Control Cardiovascular Risk in Diabetes (ACCORD) trial^3^ and the Systolic Blood Pressure Intervention Trial (SPRINT)^4^ in people with and without type 2 diabetes. SPRINT reported that aiming for a systolic blood pressure of less than 120 mm Hg, compared with less than 140 mm Hg, significantly lowered the risk of cardiovascular disease among those who did not have known type 2 diabetes at baseline.^4^ By contrast, the ACCORD trial, which used identical blood pressure lowering goals and similar interventions, reported no clear preventive benefit in people with type 2 diabetes.^3^ A subsequent aggregate data meta-analysis of randomised trials, including 100 354 people with type 2 diabetes, also reported that blood pressure-lowering treatment reduces the risk of major cardiovascular disease and all-cause death overall, but with a stronger relative effect among those with a baseline systolic blood pressure of 140 mm Hg or greater.^5^ These studies have implied that use of antihypertensives at lower blood pressure thresholds, or to lower targets, in type 2 diabetes might not be worthwhile.

The third cycle of the Blood Pressure Lowering Treatment Trialists Collaboration (BPLTTC) comprises more than 350 000 participants, allowing for simultaneous investigation of heterogeneity of effect by type 2 diabetes status and systolic blood pressure categories at baseline, using the largest known dataset of randomised participants with type 2 diabetes. In this study, we analysed individual participant-level data from major randomised controlled trials to investigate the effects of blood pressure-lowering treatment on the risk of major cardiovascular events in people with and without type 2 diabetes, as well as by baseline levels of systolic blood pressure.

## Methods

### Study setting, study design, and eligibility criteria

We performed an individual participant-level data meta-analysis using the BPLTTC dataset. The BPLTTC is an international collaboration of investigators of major pharmacological trials on blood pressure-lowering treatment, currently involving 52 randomised studies with individual-level data for 363 684 participants (December, 2021). Details of the current cycle of collaboration and the BPLTTC initial systematic review are published elsewhere.^6^, ^7^ The analysis included all trials with at least 1000 person-years of follow-up in each randomly assigned group that provided individual-level data to the collaboration and shared information on type 2 diabetes diagnosis at baseline, blood pressure levels at randomisation and during follow-up, and outcome data for cardiovascular events. Trials that were exclusively conducted on people with heart failure or short-term therapies in people with acute myocardial infarction or other acute settings were excluded. Further details on the inclusion and exclusion criteria published elsewhere.^6^, ^8^, ^9^ A study protocol was developed before releasing a dataset for statistical analysis and was finalised with extensive feedback from international collaborators and the BPLTTC steering committee. The BPLTTC obtained ethics approval from the Oxford Tropical Research Ethics Committee (OxTREC Reference 545–14). Each included trial obtained informed consent from the study participants.

### Treatment and comparison groups

We defined treatment and comparator groups in each trial on the basis of the trial design. In placebo-controlled trials, the placebo group was defined as the comparator and the active treatment group as the intervention. In head-to-head trials comparing two or more classes of drugs, the group with the greater systolic blood pressure reduction was considered the treatment group and the other treatment group (or groups) was the comparator. In trials investigating two blood pressure-lowering strategies (ie, intensive versus standard strategies), the intensive group was defined as the treatment group and the standard as the comparator group. Details of the comparison groups, participant characteristics, trial designs, and level of blood pressure reduction in each trial have been published elsewhere.^6^, ^7^, ^8^, ^9^

### Primary and secondary outcomes

The primary outcome was the occurrence of major cardiovascular events, defined as the first occurrence of fatal or non-fatal stroke or cerebrovascular disease (both ischaemic and haemorrhagic), fatal or non-fatal ischaemic heart disease, or heart failure resulting in death or hospitalisation. Secondary outcomes were the individual components of the primary outcome, as well as cardiovascular-related causes of death (including myocardial infarction, sudden cardiac, coronary heart disease, stroke, or heart failure) and all-cause mortality. The diagnostic information provided by each trial was used to define the outcomes.

### Statistical analysis

We did an intention-to-treat analysis and grouped participants according to the treatment they were initially assigned to in each trial (intervention *vs* comparator). We applied a fixed-effect, one-stage individual participant-level data meta-analysis that uses individual-level data from all trials simultaneously by fitting a single statistical model.^10^ A Cox proportional hazard model, stratified by trial, was used to estimate the hazard ratio (HR). A Poisson regression model with an identity link was used to calculate the absolute risk reduction over follow-up duration. Kaplan-Meier estimates of cumulative incidence were used to compute event rates, which were then plotted separately for the group with and without type 2 diabetes at baseline. We performed a univariate meta-regression analysis to evaluate the influence of blood pressure-lowering treatment on the proportional risk reduction of major cardiovascular disease at the trial level for individuals with and without type 2 diabetes. In this analysis, calculated blood pressure decreases between comparison groups and estimated HRs and their 95% CIs for each trial, both stratified for type 2 diabetes status at baseline, were used.

We standardised the estimates for a reduction in systolic blood pressure of 5 mm Hg, which was a close approximation to the mean blood pressure reduction reached across blood pressure-lowering intensity and placebo-controlled trials.^9^, ^11^ We included an interaction term for type 2 diabetes status and treatment in the model to assess the heterogeneity of effect by type 2 diabetes status at baseline. Furthermore, we conducted the analysis separately for people with and without type 2 diabetes, and then further assessed interaction by baseline categories of systolic blood pressure (in 10 mm Hg increments from <120 mm Hg to ≥170 mm Hg). We also investigated effects on secondary outcomes (components of major cardiovascular disease) to compare consistency of patterns. We used likelihood-ratio tests, adjusted and unadjusted for multiple comparisons, to test for interaction. Hommel's method was used to adjust the p value for multiple comparisons to reduce the possibility of false-positive findings.^12^, ^13^

We conducted several complementary and sensitivity analyses. We investigated drug class effects stratified by type 2 diabetes status to assess whether any observed heterogeneity of effects might be explained by differential use of drugs in these two groups. The effect of each of the five major blood pressure-lowering drug classes—namely, angiotensin-converting enzyme inhibitors, angiotensin II receptor blockers, β blockers, calcium channel blockers, and thiazide diuretics—was estimated using a network meta-analysis framework.^14^, ^15^ Using individual-level data from each trial, the logistic regression model was used to estimate relative risk (RR) for each available comparison, separately for those with and without type 2 diabetes. Markov chain Monte-Carlo simulation, with four chains and 100 000 iterations after a 10 000 burn-in, was used to fit the network meta-analysis model.^14^ Furthermore, we conducted analyses without standardisation for blood pressure reduction across trials. To test the validity of type 2 diabetes ascertainment at baseline, we restricted the analysis to trials that used at least one laboratory measurement for diagnosis of type 2 diabetes at baseline. We also repeated analyses, excluding head-to-head trials to assess their effect on the main results. All statistical analyses were done using R (version 4.0.2).

### Role of the funding source

The funders of the study had no role in study design, data collection, data analysis, data interpretation, or writing of the report.

## Results

Because of the absence of time-to-event information, we excluded one trial^16^ from the BPLTTC database. Data for 358 533 participants from 51 randomised clinical trials were included in the analysis (appendix p 4–12). Of 51 trials, three (6%) trials included only participants with no previous history of type 2 diabetes at the time of enrolment, 41 (80%) trials included both participants with and without type 2 diabetes at baseline, and seven (14%) trials were conducted exclusively on participants with type 2 diabetes at baseline. The mean age and percentage of women were similar between groups with and without type 2 diabetes at randomisation (table). Baseline systolic/diastolic blood pressure means were 149/84 mm Hg (SD 19/11) in participants with type 2 diabetes and 153/88 mm Hg (SD 21/12) in participants without type 2 diabetes. Participants with a history of type 2 diabetes had a higher BMI at baseline and were less likely to be current smokers (table). Peripheral vascular disease and chronic kidney disease were more often comorbid conditions among participants with type 2 diabetes at baseline, whereas other comorbidities were distributed evenly across the two groups. A larger number of individuals with type 2 diabetes than those without had a history of using diuretics, angiotensin-converting enzyme inhibitors, and angiotensin II receptor blockers before being random allocated into the trial (table).

**Table:** Baseline characteristics of participants stratified by type 2 diabetes at baseline

		**Diabetes (N=103 325)**	**No diabetes (N=255 208)**
Sex			
	Female	43 276/103 325 (41·9%)	105 832/255 198 (41·5%)
	Male	60 049/103 325 (58·1%)	149 366/255 198 (58·5%)
Age, years		65·4 (8·2)	64·8 (10·2)
Systolic blood pressure, mm Hg		149·5 (19·9)	153·5 (21·7)
Diastolic blood pressure, mm Hg		84·1 (11·5)	88·7 (12·5)
BMI, kg/m^2^		29·3 (5·5)	27·3 (8·0)
Smoking status			
	Never	21 940/48 160 (45·6%)	58 134/133 247 (43·6%)
	Past	19 193/48 160 (39·9%)	47 647/133 247 (35·8%)
	Current	6971/48 160 (14·5%)	26 741/133 247 (20·1%)
Ethnicity			
	White/Caucasian/European	51 276/84 388 (60·8%)	118 206/177 623 (66·5%)
	Black	8916/84 388 (10·6%)	16 403/177 623 (9·2%)
	Hispanic	7661/84 388 (9·1%)	13 631/177 623 (7·7%)
	Asians	13 089/84 388 (15·5%)	25 337/177 623 (14·3%)
	Other	3446/84 388 (4·1%)	4046/177 623 (2·3%)
Categories of systolic blood pressure, mm Hg			
	<120	5133/101 514 (5·1%)	11 583/255 047 (4·5%)
	120 to 129	9188/101 514 (9·1%)	20 936/255 047 (8·2%)
	130 to 139	15 686/101 514 (15·5%)	32 408/255 047 (12·7%)
	140 to 149	21 500/101 514 (21·2%)	44 582/255 047 (17·5%)
	150 to 159	18 951/101 514 (18·7%)	41 911/255 047 (16·4%)
	160 to 169	15 228/101 514 (15·0%)	45 667/255 047 (17·9%)
	≥170	15 828/101 514 (15·6%)	57 974/255 047 (22·7%)
Categories of diastolic blood pressure, mm Hg			
	<70	9209/101 512 (9·1%)	14 207/255 047 (5·6%)
	70 to 79	22 440/101 512 (22·1%)	39 841/255 047 (15·6%)
	80 to 89	35 267/101 512 (34·7%)	72 893/255 047 (28·6)
	90 to 99	24 718/101 512 (24·3%)	73 059/255 047 (28·6%)
	100 to 109	8073/101 512 (8·0%)	42 248/255 047 (16·6%)
	≥110	1805/101 512 (1·8%)	12 799/255 047 (5·0%)
Comorbidity			
	Peripheral vascular disease	4433/33 434 (13·3%)	8462/100 780 (8·4%)
	Atrial fibrillation	2942/10 3325 (2·8%)	7548/255 208 (3·0%)
	Chronic kidney disease	6980/28 484 (24·5%)	17 081/116 572 (14·7%)
	Cerebrovascular disease	14 056/73 483 (19·2%)	36 627/213 915 (17·1%)
	Ischaemic heart disease	32 567/100 380 (32·4%)	87 410/255 043 (34·3%)
Previous use of non-study medications			
	Diuretics	14 864/55 460 (26·8%)	19 554/110 374 (17·7%)
	α blockers	1674/35 999 (4·7%)	3176/82 401 (3·9%)
	β blockers	18 231/56 654 (32·2%)	41 697/118 368 (35·2%)
	Angiotensin-converting enzyme inhibitors	22 160/53 219 (41·6%)	26 198/96 734 (27·1%)
	Angiotensin receptor blockers	3759/37 143 (10·1%)	4818/63 448 (7·6%)
	Calcium channel blockers	19 265/56 654 (34·0%)	36 770/118 398 (31·1%)
	Antiplatelets	22 438/49 780 (45·1%)	28 584/68 209 (41·9%)
	Anticoagulants	1821/33 599 (5·4%)	4748/51 670 (9·2%)
	Lipid-lowering treatments	20 653/49 357 (41·8%)	33 811/98 674 (34·3%)
Follow-up, years		4·33 (3·1–5·0)	4·13 (3·0–5·0)

Data are presented as n/N (%), mean (SD), or median (IQR). Due to missing data, the number of participants (denominator) for some categorical variables differs from the total reported in the column.

**Figure 1:**
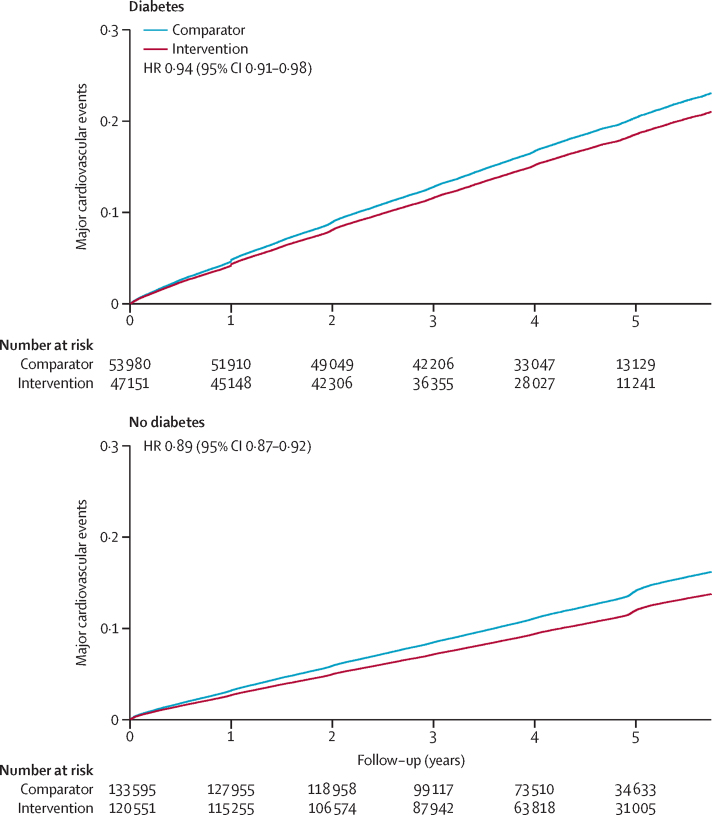
Cumulative probability of major cardiovascular events by treatment allocation per 5 mm Hg reduction in systolic blood pressure, stratified by type 2 diabetes status at baseline Major cardiovascular events are defined as a composition of fatal or non-fatal stroke, fatal or non-fatal ischaemic heart disease, or heart failure causing death or requiring hospitalisation. HR=hazard ratio.

42 931 major cardiovascular disease events occurred during 4·2 years of median follow-up (IQR 3·0–5·0). The overall numbers of events for each component of major cardiovascular diseases were 14 768 for stroke, 21 093 for ischaemic heart disease, 7908 for heart failure, 11 725 for cardiovascular-related causes of death, and 30 658 for all-cause mortality. The cumulative incidences for the primary outcome were 14·3 (95% CI 14·1–14·5) per 100 000 person-years of follow-up in participants with type 2 diabetes and 8·51 (8·4–8·6) per 100 000 person-years of follow-up in participants without type 2 diabetes. In participants with type 2 diabetes, the incidence rates of primary outcomes per 100 000 person-years of follow-up between comparator and intervention groups were 15·5 (95% CI 15·2–15·9) and 13·9 (13·6–14·3), respectively. The corresponding incidence rates in participants without type 2 diabetes at baseline were 9·3 (95% CI 9·2–9·5) and 7·8 (7·7–8·0), respectively (figure 1).

**Figure 2:**
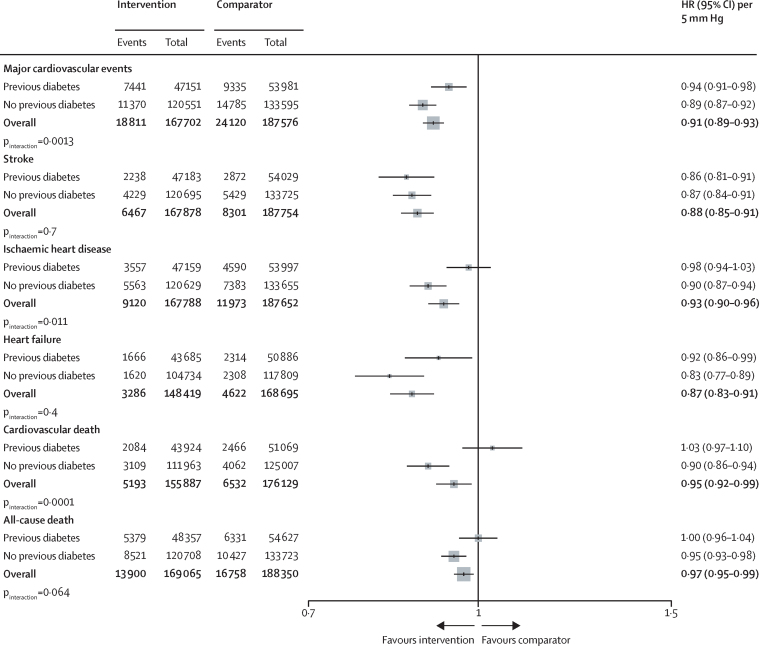
Effects of blood pressure-lowering treatment on primary and secondary outcomes, by type 2 diabetes status at baseline HRs were standardised for blood pressure reduction across trials and rescaled to a 5 mm Hg reduction in systolic blood pressure. p_interaction_ was adjusted for multiple comparisons. HR=hazard ratio.

A 5 mm Hg reduction in systolic blood pressure reduced the risk of developing a major cardiovascular event in participants with and in those without type 2 diabetes, with a weaker relative treatment effect in participants with type 2 diabetes (HR 0·94 [95% CI 0·91–0·98]) than in those without type 2 diabetes (0·89 [0·87–0·92]; p_interaction_=0·0013; Figure 1, Figure 2). The heterogeneity of effects observed for the primary outcome was mostly driven by ischaemic heart disease, for which effects were weaker in people with type 2 diabetes (HR 0·98 [95% CI 0·94–1·03]) than in those without type 2 diabetes (0·90 [0·87–0·94]; p_interaction_=0·011; figure 2). For stroke, effects appeared similar between type 2 diabetes status groups. For heart failure, despite a pattern of weaker HRs in participants with type 2 diabetes (HR 0·92 [95% CI 0·86–0·99] participants with diabetes *vs* 0·83 [0·77–0·89] participants without diabetes), there was also no statistical evidence for an interaction. For cardiovascular death and all-cause death, data suggested a heterogeneous treatment effect, with no apparent beneficial effect in people with a history of type 2 diabetes (p_interaction_<0·0001 for cardiovascular death and p_interaction_=0·064 for all-cause death; figure 2).

**Figure 3:**
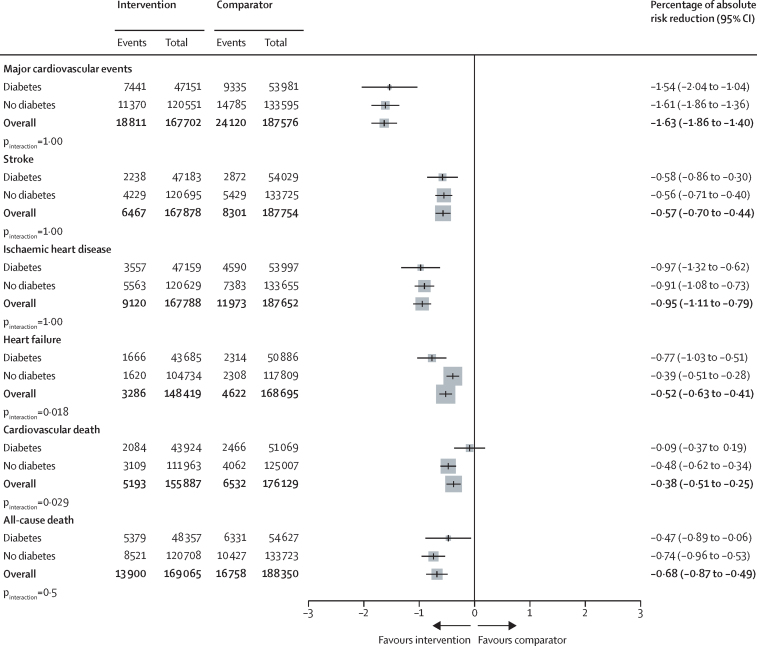
Percentage absolute risk reductions for the effect of blood pressure-lowering treatment on primary and secondary outcomes, by type 2 diabetes status at baseline Absolute risk reduction was estimated using a Poisson regression model with identity link. The unit is the percentage of absolute risk difference (treatment *vs* comparator), over follow-up time and reflects mean of blood pressure reduction across all trials. p_interaction_ was adjusted for multiple comparisons.

**Figure 4:**
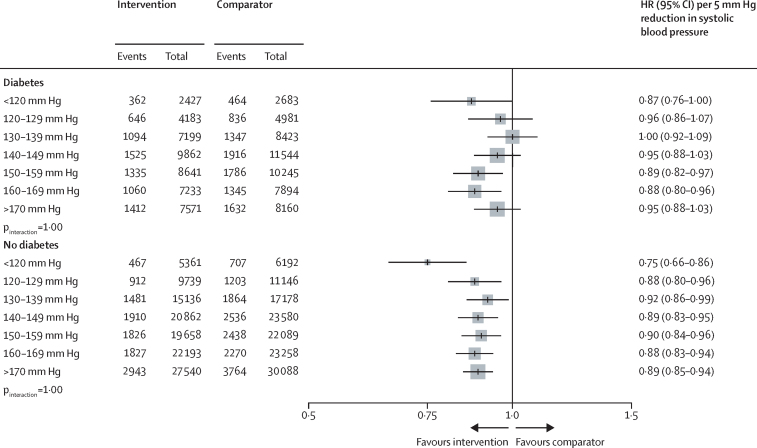
Effects of systolic blood pressure-lowering treatment on major cardiovascular events stratified by baseline systolic blood pressure and type 2 diabetes status at baseline HRs were standardised for blood pressure reduction across trials and rescaled to a 5 mm Hg reduction in systolic blood pressure. p_interaction_ was adjusted for multiple comparisons. HR=hazard ratio.

The HRs for major cardiovascular events in participants with and without type 2 diabetes were proportional to the magnitude of the systolic blood pressure reduction obtained at the trial level, but to a lesser extent in people with type 2 diabetes versus those without (appendix p 14). The observed heterogeneous relative treatment effects largely disappeared, or at least diminished, when effects were compared on the absolute risk scale (owing of the greater absolute baseline risk in participants with type 2 diabetes). However, for cardiovascular death, absolute risk reductions remained weaker among participants with type 2 diabetes versus those without (figure 3). In stratified analyses, we did not find reliable evidence for heterogeneity of treatment effects by baseline systolic blood pressure level in participants with or without type 2 diabetes, for either primary or secondary outcomes (figure 4; appendix p 15).

In keeping with the primary findings, complementary analysis using stratified network meta-analysis showed no evidence that relative treatment effects were stronger among participants with type 2 diabetes compared with those without for any of the drug classes investigated (appendix p 16). In sensitivity analysis, the findings were similar to the main results in the analysis without standardisation for blood pressure reduction across trials (appendix p 17). Furthermore, we did not find any substantial change in the treatment effects when we restricted the analysis to trials that used a laboratory test for diagnosis of type 2 diabetes at baseline (appendix p 18). No change in effect sizes were reported after excluding head-to-head trials (appendix p 13).

## Discussion

In this individual participant-level data meta-analysis of major pharmacological blood pressure-lowering trials, comprising 103 325 participants with type 2 diabetes and 255 208 without type 2 diabetes, blood pressure-lowering treatment reduced the risk of major cardiovascular events in those with and without type 2 diabetes. The relative effects, however, were weaker in participants with established type 2 diabetes than in those without. Nonetheless, because participants with type 2 diabetes were at a higher risk of major cardiovascular events, the absolute risk reductions were broadly similar between the two groups. Investigation of the underlying reasons for the heterogeneous relative effects suggested that the differences were not substantially influenced by the levels of systolic blood pressure at baseline or types of antihypertensive drugs used.

Antihypertensive therapy is an established strategy for reducing the risk of macrovascular and microvascular events in people with type 2 diabetes. The UK Prospective Diabetes Study (also known as UKPDS) was one of the first large-scale trials of antihypertensive treatment in people with type 2 diabetes and showed notable reductions in risk of cardiovascular events in the presence of hypertension.^17^ Several subsequent trials including people with type 2 diabetes assessed the effects of particular drugs or different blood pressure management strategies. For example, the ADVANCE trial suggests that adopting a fixed regimen of perindopril–indapamide reduces all-cause death and major cardiovascular events in individuals with type 2 diabetes, regardless of baseline blood pressure or 10-year cardiovascular risk.^18^ An individual participant-level data meta-analysis of these studies was reported by the BPLTTC in 2005.^19^ On the basis of data from 158 700 participants, of whom 33 395 had type 2 diabetes, the use of antihypertensive therapy was shown to reduce the risk of major cardiovascular events roughly to a similar extent in individuals with type 2 diabetes and in those without type 2 diabetes. However, the study had limited statistical power, analyses were not standardised for differing magnitudes of blood pressure lowering, and stratification by baseline blood pressure levels was not done.

Several more recent studies have aimed to investigate the differences in the magnitude of the cardioprotective effect of blood pressure lowering by baseline blood pressure. Perhaps the most surprising findings came from the ACCORD trial^3^ and SPRINT,^4^ which, despite very similar designs, came to different conclusions. This discrepancy generated the hypothesis that intensive blood pressure reduction might not be useful in people with type 2 diabetes.^20^, ^21^, ^22^ Support for this hypothesis came from conventional meta-analyses of published information on people with type 2 diabetes. One study that included 100 354 participants with type 2 diabetes reported beneficial effects of blood pressure-lowering treatment on the risk of major cardiovascular events (HR 0·89 [95% CI 0·83–0·95] per 10 mm Hg lower systolic blood pressure).^5^ However, when trials were stratified into two categories of baseline systolic blood pressure with a cutoff at 140 mm Hg, substantial heterogeneity was observed; in the group with a baseline systolic blood pressure of less than 140 mm Hg, there was no clear reduction in the risk of cardiovascular events (HR 0·96 [95% CI 0·88–1·05]).^5^ Another conventional meta-analysis combining data from 73 738 participants with diabetes showed that antihypertensive treatment reduced the risk of mortality and cardiovascular morbidity in people with type 2 diabetes and a systolic blood pressure of more than 140 mm Hg, but when baseline systolic blood pressure was lower than 140 mm Hg, treatment was more likely to cause harm than benefit, largely owing to an excess risk of cardiovascular death in the treated group.^23^ However, these meta-analyses had no individual-level information and, therefore, their findings could be subject to ecological bias.^24^ Further, some of the differences between these studies —namely, the selection of studies, the methods of weighting the studies, and the grouping of the studies and participants—might have contributed to the discrepant findings. Future studies could explore the importance of these features further. Using a large-scale database of randomised clinical trials of participants with and without type 2 diabetes, we were able to directly compare the effects of a fixed level of blood pressure reduction in people with and without type 2 diabetes. Additionally, we were able to stratify analyses by precise categories of baseline blood pressure.

Our findings showed that, although blood pressure-lowering treatment reduced the risk of major cardiovascular events in people with type 2 diabetes, the amount of the relative risk reduction was slightly smaller in those with type 2 diabetes than in those without type 2 diabetes, with no apparent treatment effect on ischaemic heart disease, cardiovascular-related death, and all-cause death. Differing pathophysiological mechanisms might be associated with the variation in effects in people with type 2 diabetes versus those without. Evidence exists that type 2 diabetes itself is an important risk factor for cardiovascular disease.^25^ A previous BPLTTC study that used randomised trials and genetic information reported consistent evidence that blood pressure-lowering treatment is associated with a lower risk of type 2 diabetes.^15^ As a result, the effects of blood pressure-lowering treatment on the risk of cardiovascular events might, in part, operate through reduction of type 2 diabetes risk. If true, the diluted magnitude of effect in people with type 2 diabetes might be explained partially by the mediator role of type 2 diabetes, which will not operate in people who already have established type 2 diabetes. However, this study on its own cannot explain the biological reasons behind the differences in effect or why the effects for some subtypes of cardiovascular disease like stroke were largely consistent in people with or without type 2 diabetes.

Given that the background risk of cardiovascular disease was higher in participants with type 2 diabetes than in those without, absolute risk reductions were broadly similar in both groups. If it is assumed that people with type 2 diabetes in routine practice are also at a very high risk of cardiovascular diseases, then our findings would mean that, despite the weaker relative risk reductions, people with type 2 diabetes would have much to gain from even modest blood pressure reduction. However, trial data are rarely representative of the population to whom the results are applied. The risk of cardiovascular diseases or death depends on a number of factors and could vary substantially among individuals with type 2 diabetes. For instance, the adoption of screening programmes has led to an increase in the number of individuals diagnosed with type 2 diabetes but with much lower average risks of cardiovascular diseases and death than previously reported.^26^ Therefore, we caution against overgeneralisation of the absolute risk reductions from the randomised controlled trials and recommend incorporation of risk stratification at the point of clinical decision making for more meaningful estimation of the absolute gains from treatment and the selection of individuals most likely to benefit from them.

Our study further showed no heterogeneity of effects by baseline categories of systolic blood pressure. Although the relative effects on cardiovascular outcomes per unit reduction in blood pressure were shown to be weaker in type 2 diabetes, this does not imply that blood pressure-lowering treatment ceased to be effective or was even harmful at certain blood pressure thresholds. Therefore, previous calls for adoption of any blood pressure thresholds for use of antihypertensive therapy^20^, ^21^, ^22^ are challenged by our study. Clinicians caring for people with type 2 diabetes should inform the individuals that antihypertensive therapy affords cardiovascular disease risk reduction that is proportional to the degree of blood pressure reduction and irrespective of their measured blood pressure.

Weaker relative risk reductions in participants with type 2 diabetes seem surprising when considering that other disease phenotypes have not previously shown this pattern. In two BPLTTC studies, for instance, relative effects did not vary by presence or absence of cardiovascular diseases or atrial fibrillation.^9^, ^11^ Thus, this finding raises questions about biological or even statistical reasons underlying the heterogenous effect in type 2 diabetes. To further investigate the findings, we conducted several supplementary analyses. We performed a meta-regression stratified by type 2 diabetes and a network meta-analysis by drug classes. These analyses supported the robustness of our findings and provided no evidence that perhaps differing types of drug classes might explain the observed heterogeneity. Another possible reason is that higher average risk in type 2 diabetes might have diluted the relative contribution of blood pressure reduction. However, this reason also seems unlikely given that in an earlier BPLTTC study, stratification by baseline cardiovascular clinical risk did not modify relative effects.^27^ In other studies, people with cardiovascular diseases and atrial fibrillation were also at higher average risk but no heterogeneous treatment effects were reported.^9^, ^11^

Some limitations should be noted when interpreting and generalising our results. We acknowledge that type 2 diabetes itself can be a heterogeneous condition. In the trials included, type 2 diabetes was ascertained at the start of the trials using a range of criteria. No substantial differences were found in a sensitivity analysis when the main results were stratified on the basis of alternative type 2 diabetes ascertainment methods. Nonetheless, whether our findings apply to different stages of disease requires further investigation. A post-hoc analysis of the SPRINT trial,^28^ which stratified participants without known type 2 diabetes by baseline fasting serum glucose concentration, suggested that intensive blood pressure-lowering treatment might have a similar beneficial effect on major cardiovascular events and all-cause mortality in participants with prediabetes versus those with normal blood glucose concentrations, and this effect could be consistent across the fasting serum glucose spectrum at baseline; however, CIs were wide. However, we know that in prediabetes, the cardiovascular risk is not escalated beyond usual risk factors, whereas it is escalated in diabetes.^29^ Further studies are required to investigate effects in more detail across the range of glucose intolerance and diabetes, and in different management strategies for glycaemia in type 2 diabetes. Relatedly, the duration of diabetes and, in particular, concurrent complications, such as nephropathy or chronic kidney disease, could have a role or explain the heterogeneous effects. Chronic kidney disease is a common long-term complication of diabetes, and the magnitude of blood pressure reduction in participants without chronic kidney disease is greater than in those with some degree of chronic kidney disease, with a heterogeneity of blood pressure treatment effect observed between these groups.^30^ Future BPLTTC projects will investigate the contribution of these factors and might help to refine patient identification and treatment recommendations. Furthermore, there is evidence to suggest that blood pressure reduction in people with type 2 diabetes reduces the risk of microvascular events.^5^ We did not investigate these outcomes in the current analysis, which will be the subject of a future study. Finally, newer classes of drugs licensed for use in people with diabetes, such as GLP-1 receptor agonists and SGLT2 inhibitors, reduce blood pressure and the risk of cardiovascular events.^31^ Although their use in the participants from the BPLTTC trial was scarce, these drugs are increasingly used in clinical practice. Given their differing pathways of action from classic antihypertensive drugs investigated in our study, we expect their effects on cardiovascular disease risk reduction to be complementary. However, future studies could investigate this further.

This study shows that the relative effect of blood pressure reduction on major cardiovascular events is weaker in people with type 2 diabetes than in people without type 2 diabetes. However, this result was not because lowering blood pressure to below a certain threshold was ineffective or harmful. Indeed, across the full spectrum of baseline blood pressure categories, there was no subgroup in which harmful effects on major cardiovascular outcomes were detected. Despite the weaker relative effect in type 2 diabetes, in populations included in previous randomised controlled trials the absolute risk reductions were broadly similar in those with type 2 diabetes and those without. These findings emphasise the importance of blood pressure lowering for cardioprotection before the onset of type 2 diabetes. In people with established type 2 diabetes, the current blood pressure thresholds for initiation of blood pressure treatment do not seem to be justified. This study calls for the removal of specific blood pressure thresholds when selecting people with type 2 diabetes for antihypertensive therapy.

## Data sharing

The governance of the BPLTTC has been reported previously. The BPLTTC is governed by the University of Oxford's policies on research integrity and codes of practice and follows the university's policy on the management of research data and records. Scientific activities based on the BPLTTC dataset are overseen by the BPLTTC steering committee. All data shared with the BPLTTC will be considered confidential and will not be provided to any third party. Requests for data should be made directly to the data custodians of individual trials. Information about individual projects is posted online.

## Declaration of interests

KR reports personal fees from the BMJ Heart and PLOS Medicine, outside of the submitted work. MW reports personal funding from Amgen, Kyowa Kirin, and Freeline, outside of the current work. NS has consulted for, or received lecture fees from, Afimmune, Amgen, AstraZeneca, Boehringer Ingelheim, Eli Lilly, Hanmi Pharmaceuticals, MSD, Novartis, Novo Nordisk, Pfizer, and Sanofi; and received grant support from AstraZeneca, Boehringer Ingelheim, Novartis, and Roche Diagnostics through his institution, the University of Glasgow. RRH reports research support from AstraZeneca, Bayer, and MSD; and personal fees from Anji Pharmaceuticals, Bayer, Novartis, and Novo Nordisk. RJM received blood pressure monitors from Omron for research and his institution receives fees from Omron and Sensyne for blood pressure telemonitoring systems. JC reports research grants and personal fees from Servier for the ADVANCE and PROGRESS trials, and grants from the National Health and Medical Research Council of Australia for these two trials. All other authors declare no competing interests.
