# Supplementary material for: Blood pressure-lowering treatment for prevention of major cardiovascular diseases in people with and without type 2 diabetes: an individual participant-level data meta-analysis
Source: Lancet Diabetes Endocrinol. 2022 Sep;10(9):645–54. doi: 10.1016/S2213-8587(22)00172-3 (PMC9622419; doi:10.1016/S2213-8587(22)00172-3)
Supplement: Supplementary appendix [file mmc1.pdf]

THE LANCET
Diabetes & Endocrinology

Supplementary appendix

This appendix formed part of the original submission and has been peer reviewed. We post it as supplied by the authors.

Supplement to: Nazarzadeh M, Bidel Z, Canoy D, et al. Blood pressure-lowering treatment for prevention of major cardiovascular diseases in people with and without type 2 diabetes: an individual participant-level data meta-analysis. *Lancet Diabetes Endocrinol* 2022; published online July 22. https://doi.org/10.1016/S2213-8587(22)00172-3.

Supplementary materials

Blood pressure-lowering treatment for prevention of major cardiovascular diseases in people with and without type 2 diabetes: an individual participant-level data meta-analysis

Contents

Working group 2

The Blood Pressure Lowering Treatment Trialists' Collaboration 2

Table S1. General characteristics of trials included in the analysis. 4

Table S2. Sensitivity analysis excluding head-to-head trials for the effect of blood pressure-lowering treatment on primary and secondary outcomes, by type 2 diabetes status at baseline. 13

Figure S1. Meta-regression of intensity of blood pressure reduction and hazard ratio of major cardiovascular events, by type 2 diabetes status at baseline. 14

Figure S2. Effects of blood pressure-lowering treatment on primary and secondary outcomes stratified by baseline systolic blood pressure and type 2 diabetes at baseline. 15

Figure S3. Effect of major antihypertensive drug classes on the risk major cardiovascular outcomes, by type 2 diabetes at baseline. 16

Figure S4. The unstandardised effects of blood pressure-lowering treatment on primary and secondary outcomes, by type 2 diabetes status at baseline. 17

Figure S5. Sensitivity analysis restricted to trials that used a laboratory test for diagnosis of type 2 diabetes at baseline. 18

References 19

Working group

Milad Nazarzadeh, Zeinab Bidel, Dexter Canoy, Emma Copland, Derrick A Bennett, Abbas Dehghan, George Davey Smith, Rury R. Holman, Mark Woodward, Ajay Gupta, Amanda Adler, Malgorzata Wamil, Naveed Sattar, William C. Cushman, Richard J McManus, Koon Teo, Barry R Davis, John Chalmers, Carl J. Pepine, Kazem Rahimi

The Blood Pressure Lowering Treatment Trialists' Collaboration

Steering Committee: Kazem Rahimi (Chair), Koon Teo, Barry R Davis, John Chalmers, Carl J Pepine

Collaborating Trialists: A Adler (UKPDS [UK Prospective Diabetes Study]), L Agodoa (AASK [African-American Study of Kidney Disease and Hypertension]), A Algra (Dutch TIA Study [Dutch Transient Ischemic Attack Study]), F W Asselbergs (PREVEND-IT [Prevention of Renal and Vascular End-stage Disease Intervention Trial]), N Beckett (HYVET [Hypertension in the Very Elderly Trial]), E Berge (deceased) (VALUE trial [Valsartan Antihypertensive Long-term Use Evaluation trial]), H Black (CONVINCE [Controlled Onset Verapamil Investigation of Cardiovascular End Points]), E Boersma (EUROPA [European trial on reduction Of cardiac events with Perindopril among patients with stable coronary Artery disease]), F P J Brouwers (PREVEND-IT), M Brown (INSIGHT [International Nifedipine GITS Study: Intervention as a Goal in Hypertension]), J Brugts (EUROPA),C J Bulpitt (EWPHE [European Working Party on High Blood Pressure in the Elderly], HYVET), R P Byington (PREVENT [Prospective Randomized Evaluation of the Vascular Effects of Norvasc Trial]), J Chalmers (ADVANCE [Action in Diabetes and Vascular Disease: Preterax and Diamicron MR Controlled Evaluation], PROGRESS [Perindopril protection against recurrent stroke]), W C Cushman (ACCORD [Action to Control Cardiovascular Risk in Diabetes], ALLHAT [Antihypertensive and Lipid-Lowering Treatment to Prevent Heart Attack Trial], SPRINT [Systolic Blood Pressure Intervention Trial]), J Cutler (ALLHAT), B R Davis (ALLHAT), R B Devereaux (LIFE [Losartan Intervention For Endpoint reduction in hypertension]), J P Dwyer (IDNT [Irbesartan Diabetic Nephropathy Trial]), R Estacio (ABCD [Appropriate Blood Pressure Control in Diabetes]), R Fagard (Syst-Eur [SYSTolic Hypertension in EURope]), K Fox (EUROPA), T Fukui (CASE-J [Candesartan Antihypertensive Survival Evaluation in Japan]), A K Gupta (ASCOT-BPLA [AngloScandinavian Cardiac Outcomes Trial—Blood Pressure Lowering Arm]), R R Holman (UKPDS), Y Imai (HOMED-BP [Hypertension Objective Treatment Based on Measurement by Electrical Devices of Blood Pressure]), M Ishii (JMIC-B [Japan Multicenter Investigation for Cardiovascular Diseases-B]), S Julius (VALUE), Y Kanno (E-COST [Efficacy of Candesartan on Outcome in Saitama Trial]), S E Kjeldsen (VALUE, LIFE), J Kostis (SHEP [Systolic Hypertension in the Elderly Program]), K Kuramoto (NICS-EH [National Intervention Cooperative Study in Elderly Hypertensives]), J Lanke (STOP Hypertension-2 [Swedish Trial in Old Patients with Hypertension-2], NORDIL [Nordic Diltiazem]), E Lewis (IDNT), J B Lewis (IDNT), M Lievre (DIABHYCAR [Non-insulin-dependent diabetes, hypertension, microalbuminuria or proteinuria, cardiovascular events, and ramipril study]), L H Lindholm (CAPPP [Captopril Prevention Project], STOP Hypertension-2, NORDIL), S Lueders (MOSES [The Morbidity and Mortality After Stroke, Eprosartan Compared With Nitrendipine for Secondary Prevention]), S MacMahon (ADVANCE, PART-2 [Prevention of Atherosclerosis with Ramipril Trial]), G Mancia (INSIGHT), M Matsuzaki (COPE [The Combination Therapy of Hypertension to Prevent Cardiovascular Events]), M H Mehlum (VALUE), S Nissen (CAMELOT [Comparison of Amlodipine vs Enalapril to Limit Occurrences of Thrombosis]), H Ogawa (HIJ-CREATE [Heart Institute of Japan Candesartan Randomized Trial

for Evaluation in Coronary Heart Disease]), T Ogihara (CASE-J, COLM [Combinations of OLMesartan], COPE), T Ohkubo (HOMED-BP), C R Palmer (INSIGHT), A Patel (ADVANCE), C J Pepine (INVEST [International Verapamil SR-Trandolapril Study]), M A Pfeffer (PEACE [Prevention of Events With Angiotensin- Converting Enzyme Inhibition]), B Pitt (PREVENT), N R Poulter (ASCOT), H Rakugi (CASE-J, VALISH [Valsartan in Elderly Isolated Systolic Hypertension Study]), G Reboldi (Cardio-Sis [CARDIOvascolari del Controllo della Pressione Arteriosa SIStolica]), C Reid (ANBP2 [The Second Australian National Blood Pressure Study]), G Remuzzi (BENEDICT [BErgamo NEphrologic DIabetes Complications Trial]), P Ruggenenti (BENEDICT), T Saruta (CASE-J), J Schrader (MOSES), R Schrier (deceased) (ABCD), P Sever (ASCOT-BPLA), P Sleight (deceased; CONVINCE, HOPE [Heart Outcomes Prevention Evaluation], ONTARGET [Ongoing Telmisartan Alone and in Combination with Ramipril Global Endpoint Trial], TRANSCEND [Telmisartan Randomised AssessmeNt Study in ACE iNtolerant subjects with cardiovascular Disease]), J A Staessen (Syst-Eur), H Suzuki (E-COST), L Thijs (Syst-Eur), K Ueshima (CASE-J, VALISH), S Umemoto (COPE), W H van Gilst (PREVEND-IT), P Verdecchia (Cardio-Sis), K Wachtell (LIFE), P Whelton (SPRINT), L Wing (ANBP2), M Woodward (ADVANCE, PROGRESS), Y Yui (JMIC-B), S Yusuf (HOPE, ONTARGET, TRANSCEND), A Zanchetti (deceased; ELSA [European Lacidipine Study on Atherosclerosis], VHAS [Verapamil in Hypertension and Atherosclerosis Study]), and Z Y Zhang (Syst-Eur).

Other members: C Anderson, C Baigent, B M Brenner, R Collins, D de Zeeuw, J Lubsen, E Malacco, B Neal, V Perkovic, A Rodgers, P Rothwell, G Salimi-Khorshidi, J Sundström, F Turnbull, G Viberti, and J Wang.

Correspondence to

Prof Kazem Rahimi, Deep Medicine, Nuffield Department of Women's and Reproductive Health, University of Oxford, Oxford OX1 2BQ, UK, Email: kazem.rahimi@wrh.ox.ac.uk

Table S1. General characteristics of trials included in the analysis.

Number	Trial name	Type of trial	Inclusion criteria	Exclusion criteria	Age (years) mean (SD)	Mean of follow-up duration (year)	Intervention (n)	Comparator (n)	Number of participants with and without diabetes at baseline (with diabetes/without diabetes)	Definition of diabetes at baseline	SBP difference (mmHg) excluding first 12 months *	DBP difference (mmHg) excluding first 12 months*
1.	AASK[1]	Intensive	Age 18-70 years, African American, hypertension, renal disease (GFR=20-65 ml/min per 1.73m^2)	DBP <95 mmHg, diabetes, urine protein:creatinine ratio >25, recent malignant hypertension, secondary hypertension, non-blood pressure-related CKD, serious systemic disease, HF	54 (11)	4.8	More intensive (540)	Less intensive (554)	0/1094	Fasting glucose level ≥ 140 mg/dl (7.8 mmol/L), a random glucose level ≥ 200 mg/dl (11.1 mmol/L), or pharmacological glucose-lowering therapy	13.0	7.9
2.	ABCD[2]	Intensive	Age 40-74 years, type 2 diabetes mellitus, DBP ≥80 mmHg, not on antihypertensive treatment	Recent CAD or CeVD, heart failure, renal disease	58 (8)	4.7	More intensive (474)	Less intensive (476)	950/0	Diagnosis/history of type 2 diabetes mellitus	7.7	6.9
3.	ACCORD[3]	Intensive	Age ≥40 years with CVD or ≥50 years with substantial atherosclerosis, diagnosis/history of type 2 diabetes mellitus, HbA1c ≥7.5%, albuminuria, LVH or ≥2 CVD risk factors (dyslipidaemia, hypertension, smoking, obesity); SBP 130-180 mmHg and taking ≤3 antihypertensive drugs, 24-hour protein excretion rate <1g	Body mass index ≥45 kg/m2, serum creatinine ≥132.6 μmol/l and other serious illness	63 (7)	4.7	More intensive (2362)	Less intensive (2371)	4733/0	Diagnosis/history of type 2 diabetes mellitus, or HbA1c ≥ 7.5%	13.9	1.8
4.	ACTIVE I[4]	Placebo-controlled	Atrial fibrillation, ≥1 risk factor (age ≥75 years, on antihypertensive treatment, history of stroke, TIA or non-CNS embolism, LVEF <45%, PVD, or age 55-74 years with either CAD or diabetes)	Use of anticoagulant, peptic ulcer disease in past 6 months, history of intracerebral haemorrhage, thrombocytopaenia or mitral stenosis	70 (10)	4.1	ARB (3058)	Placebo (3076)	1120/5014	Diagnosis/history of type 2 diabetes mellitus, or pharmacological glucose-lowering therapy	2.6	1.4
5.	ADVANCE[5]	Placebo-controlled	Age ≥55 years, type 2 diabetes	Definite indication for, or contraindication to,	66 (6)	4.2	ACEI and Diuretic	Placebo (5571)	11140/0		5.4	2.1

			mellitus (diagnosed aged ≥30 years), ≥1 major CVD or ≥1 CVD risk factor (microvascular disease, smoking, dyslipidaemia, microalbuminuria, Diagnosis of type 2 diabetes mellitus for ≥10 years, age ≥65 years)	any of the study treatments or the HbA1c target ≤6·5%, indication for long-term insulin therapy at baseline			(5569)			Diagnosis of type 2 diabetes mellitus at ≥ 30 years old, or previous diagnosis of type 2 diabetes mellitus for ≥ 10 years		
6.	ALLHAT[6]	Drug classes comparison	Age ≥55 years, stage 1 or 2 hypertension plus ≥1 risk factor (MI or stroke >6 months, LVH, diagnosis/history of type 2 diabetes mellitus, smoking, HDL <0.91 mmol/l), other atherosclerotic CVD	Symptomatic or hospitalisation for HF, LVEF <35%	67 (8)	4.8	Diuretic (15255)	ACEI, CCB and Alpha-blockers (27163)	16575/25843	Diagnosis/history of type 2 diabetes mellitus, or baseline fasting glucose level of ≥126 mg/dL (7 mmol/L)	2.0	0.1
7.	ANBP[7]	Placebo-controlled	Age 30-69 years with mild hypertension (DBP 95-110 mmHg and SBP <200 mmHg)	Antihypertensive treatment in past 3 months, recent angina or MI, stroke, hormone therapy, asthma, diabetes, gout, serious disease, tricyclic antidepressant use	50 (9)	3.6	Diuretic (1721)	Placebo (1706)	0/3427	Diagnosis/history of type 2 diabetes mellitus	7.5	3.8
8.	ANBP2[8]	Drug classes comparison	Age 65-84 years, SBP ≥160 mmHg or DBP ≥90 mmHg (if SBP ≥140 mmHg), no recent CVD	Serious illness, plasma creatinine >221 µmol/l, malignant hypertension, dementia	73 (5)	4.1	Diuretic (3039)	ACEI (3044)	402/5681	Diagnosis/history of type 2 diabetes mellitus	0.9	0
9.	ASCOT-BPLA[9]	Drug classes comparison	Age 40-79 years, untreated (SBP ≥160 or DBP ≥100 mmHg) or treated hypertension (SBP ≥140 or DBP ≥90 mmHg), ≥3 CVD risk factors (documented LVH, abnormal ECG, type 2 diabetes mellitus, PAD, previous stroke or TIA, male sex, age ≥55 years, microalbuminuria or proteinuria,	Previous MI, current treatment for angina, recent CeVD, fasting triglycerides >4.5 mmol/l, heart failure, arrhythmia, haematological or biochemical abnormality at screening	63 (9)	5.3	CCB-based (9639)	Beta-blocker based (9618)	5145/14112	Fasting glucose level ≥ 126 mg/dL (7 mmol/L), or a 2-h post-load plasma of 199.8 mg/dL (11.1 mmol/l), or pharmacological and non-pharmacological glucose-lowering therapy, or diagnosis/history of type 2 diabetes mellitus	2.2	2

			smoking, TC:HDL ≥6, family history of premature coronary heart disease									
10.	BENEDICT[10]	Placebo-controlled	Age ≥40 years, untreated SBP ≥130 / DBP ≥85 mmHg or needing treatment to attain below these levels, type 2 diabetes mellitus for <25 years, urinary albumin excretion rate <20 mcg/min, serum creatinine ≤133 µmol/l	HbA1c ≥11%, nondiabetic renal disease	62 (8)	3.1	ACEI, CCB and ACEI/CCB (907)	Placebo (302)	1209/0	History of type 2 diabetes mellitus not exceeding 25 years	2	1.3
11.	CAMELOT[11]	Placebo-controlled	Age 30-79 years, coronary artery stenosis >20% by angiography, DBP <100 mmHg	Left middle coronary artery obstruction >50%, LVEF <40%, heart failure	58 (10)	1.6	CCB and ACEI (1340)	Placebo (657)	439/1542	Diagnosis/history of type 2 diabetes mellitus, or fasting glucose level ≥ 126 mg/dL (7 mmol/L)	5.3	3.3
12.	CAPPP[12]	Drug classes comparison	Age 25-66 years, DBP ≥100 mmHg on two occasions	Secondary hypertension, serum creatinine >150 µmol/l, condition requiring beta-blocker treatment	52 (8)	5.8	Beta-blocker and/or Diuretic (5493)	ACEI (5492)	572/10413	At least two abnormal fasting glucose values that were unequivocal (i.e., between 99 mg/dL [5.5 mmol/L] and 120.6 mg/dL [6.7 mmol/L]). If they were not unequivocal, diagnosis was confirmed by an oral glucose tolerance test.	2.2	1.3
13.	CARDIO-SIS[13]	Intensive	Age ≥55 years, SBP ≥150 mmHg, taking antihypertensive drug ≥12 weeks, ≥1 CV risk factor (smoking, dyslipidaemia, family history of premature CVD, prior TIA or stroke, established CAD or PAD	Fasting blood glucose ≥126 mg/dL (≥7 mmol/l), known diabetes, serious conditions, renal disease, valvular heart disease, left ventricular hypertrophy, atrial fibrillation, substance misuse	67 (7)	4.7	More intensive (558)	Less intensive (553)	0/1111	Fasting blood glucose ≥126 mg/dL (≥7 mmol/l), diagnosis/history of diabetes	3.8	1.5
14.	CASE-J[14]	Drug classes comparison	Age 20-85 years, ≥1 high-risk factor: SBP ≥180 or DBP ≥110 mmHg, type 2 diabetes mellitus, history of angina pectoris, MI, stroke, TIA >6 months	BP ≥200/120 mmHg, type 1 diabetes, HF, LEF <40%, atrial fibrillation, cancer	64 (11)	3.1	CCB (2349)	ARB (2354)	2018/2685	Fasting blood glucose ≥126 mg/dL [7 mmol/l], casual blood glucose ≥200 mg/dL [11.1 mmol/l], haemoglobin A1c ≥ 6.5%, 2-h blood glucose on 75 g oral glucose tolerance test ≥200 mg/dL [11.1 mmol/l], or pharmacological glucose-lowering therapy	1.7	0.9

15.	COLM[15]	Drug classes comparison	Age 65-84 years, hypertension (treated: blood pressure ≥140/90 mmHg; untreated: blood pressure ≥160/100 mmHg), CVD history or CVD risk factors including diabetes and dyslipidaemia	Secondary/malignant hypertension, recent major CVD, revascularisation, angina pectoris hospitalisation or severe heart failure, atrial fibrillation, hepatic or renal dysfunction	74 (5)	3.0	ARB and Diuretic (2573)	ARB and CCB (2568)	1362/3779	Diagnosis/history of type 2 diabetes mellitus, fasting blood glucose ≥110 mg/dL [6.1 mmol/l] or postprandial blood glucose≥140 mg/dL [7.8 mmol/l]	0.3	0.4
16.	CONVINCE[16]	Drug classes comparison	Age ≥55 years, hypertension, ≥1 CVD risk factor (e.g., diabetes, smoking)	HF, dysrhythmia, secondary hypertension, recent MI or stroke, renal disease, other serious disease, BP ≥190/110 mmHg without treatment	66 (7)	2.8	CCB (8179)	Beta-blocker or Diuretic (8297)	3239/13144	Diagnosis/history of type 2 diabetes mellitus	0.0	0.7
17.	COPE[17]	Drug classes comparison	Age 40-85 years, blood pressure ≥140/90 mmHg	SBP ≥200 or DBP ≥120 mmHg, secondary hypertension, type 1 diabetes or type 2 diabetes requiring insulin treatment, recent CVD or revascularisation, heart failure, atrial fibrillation/flutter, hepatic or renal dysfunction, congenital or rheumatic heart disease, cancer	64 (11)	3.6	CCB/Diuretic and CCB/ Beta-blocker (2183)	CCB and ARB (1110)	498/2795	Diagnosis/history of type 2 diabetes mellitus (excluding patients required insulin treatment)	0.4	0.4
18.	DIABHYCAR[18]	Placebo-controlled	Age ≥50 years, type 2 diabetes mellitus, urinary albumin excretion ≥20 mg/l in two consecutive urine samples	Serum creatinine >150 μmol/l, use of insulin, ACEI or ARB, heart failure, recent MI, urinary tract infection	65 (8)	3.9	ACEI (2443)	Placebo (2469)	4912/0	Treatment with at least one oral antidiabetic agent	0.9	0.4
19.	Dutch TIA Trial[19]	Placebo-controlled	TIA or non-disabling ischaemic stroke (Rankin Scale ≤3) in past 3 months	Cerebral ischaemia from identifiable causes other than arterial thrombosis or embolism	64 (10)	2.3	Beta-blocker (732)	Placebo (741)	97/1376	Diagnosis/history of diabetes, the use of oral antidiabetic drugs or insulin, or a nonfasting plasma glucose level of ≥ 199.8 mg/dl [11.1 mmol/l]	3.1	2.0
20.	ELSA[20]	Drug classes comparison	Age 45-79 years, blood pressure 150-210/95-115 mmHg	Recent MI or stroke, and type 2 diabetes mellitus	57 (7)	3.4	CCB (1177)	Beta-blocker (1157)	156/2178	Fasting plasma glucose ≥126 mg/dl [7 mmol/l], or report of current drug treatment for diabetes	0.8	0.4
21.	EUROPA[21]	Placebo-controlled	Age ≥18 years, documented MI >3 months before screening, revascularisation >6 months before screening, >70% coronary obstruction	HF, hypotension, uncontrolled hypertension, renal insufficiency, serum potassium >5.5 mmol/L	61 (9)	4.2	ACEI (6110)	Placebo (6108)	1502/10716	Diagnosis/history of diabetes, or taking antidiabetic agents	4.6	2.2
22.	EWPHE[22]	Placebo-controlled	Age ≥60 years, blood pressure 160-239/90-119 mmHg	Curable causes of high BP, retinopathy, heart failure, stroke history, hepatitis/cirrhosis, gout, malignancy, diabetes requiring insulin treatment	71 (8)	4.6	Diuretic (416)	Placebo (424)	91/734	Fasting plasma glucose ≥126 mg/dl [7 mmol/l], or diagnosis/history of type 2 diabetes mellitus	22.4	9.5

23.	HIJ-CREATE[23]	Drug classes comparison	Age 20-80 years, CAD hospitalisation and hypertension (blood pressure ≥140/90 mmHg or antihypertensive treatment use)	Secondary hypertension, recent AMI or CeVD, severe aortic valve stenosis, cardiomyopathy, serum creatinine >2 mg/dl, serum potassium >5 mmol/l, hepatic dysfunction, malignancy	65 (9)	4.0	ARB (1024)	non-ARB (1025)	1009/1040	Fasting blood glucose ≥126 mg/dL [7 mmol/l] or treatment with hypoglycaemic agents at the time of enrolment.	0.4	0.5
24.	HOMED-BP[24]	Intensive	Self-measured SBP 135-179 mmHg or DBP 85-119 mmHg, but not if DBP <65 mmHg or SBP <110 mmHg (clinic SBP <220 mmHg and DBP <125 mmHg)	None specified	60 (10)	4.9	More intensive (1759)	Less intensive (1759)	531/2915	Fasting blood glucose ≥126 mg/dL [7 mmol/l], or an HbA1c ≥ 6.5%, or treatment with oral antidiabetic drugs or insulin	2.0	0.9
25.	HOPE[25]	Placebo-controlled	Age ≥55 years, CAD, stroke, PVD or diabetes, plus ≥1 risk factor (hypertension, dyslipidaemia, smoking, or documented microalbuminuria)	HF, LEF <40%, using ACEI or vitamin E, uncontrolled hypertension, nephropathy, or recent MI or stroke	66 (7)	4.5	ACEI (4645)	Placebo (4652)	3577/5720	Diagnosis/history of type 2 diabetes mellitus	3.0	1.4
26.	HYVET[26]	Placebo-controlled	Age ≥80y years, sustained SBP ≥160 mmHg	Accelerated or secondary hypertension, recent haemorrhagic stroke, HF, serum creatinine >150 μmol/l, serum potassium <3.5 or >5.5 mmol/l, gout, and dementia	84 (3)	2.1	Diuretic (1933)	Placebo (1912)	388/3457	Diagnosis/history of type 2 diabetes mellitus, the receipt of antidiabetic treatment, or a random blood glucose > 200 mg/dl [11.1 mmol/l]	13.1	5.1
27.	IDNT[27]	Placebo-controlled	Age 30-70 years, type 2 diabetes, hypertension (blood pressure ≥135/85 mmHg or taking anti-hypertensive drug), proteinuria, serum creatinine (µmol/l): 88 to 265 (women) or 106 to 265 (men)	Age of onset type 2 diabetes mellitus <20 years, type 1 diabetes mellitus, absolute requirement for an ACEI, ARB or CCB, cardiovascular disease (unstable angina, myocardial infarction, coronary artery bypass graft surgery or percutaneous transluminal coronary angioplasty within 3 months of study entry, New York Heart Association class III or IV HF, transient ischaemic attack within 6 months of study entry, stroke within 3 months of study entry), and serum potassium outside of normal range	59 (8)	2.6	ARB and CCB (1143)	Placebo (568)	1711/0	Diagnosis/history of type 2 diabetes mellitus	2.8	2.8
28.	INSIGHT[28]	Drug classes comparison	Age 55-80 years, hypertensive (SBP ≥150 or DBP ≥95 mmHg, or SBP ≥160 mmHg), ≥1 other risk factor (TC ≥6.43 mmol/l, smoking, family history of premature	None specified	65 (6)	2.8	Diuretic (3164)	CCB (3157)	1302/5019	Diagnosis/history of diabetes mellitus	1.1	0.9

			MI, CAD, other CVD									
29.	INVEST[29]	Drug classes comparison	Age ≥50 years, documented CAD, essential hypertension requiring drug therapy, heart failure Class I-III	Patients taking beta-blocker within two weeks of randomization or for recent MI	66 (10)	2.8	CCB (10648)	non-CCB (10672)	5879/15441	History of or currently taking antidiabetic medications	0.1	0.2
30.	JMIC-B[30]	Drug classes comparison	Age <75 years, hypertension (blood pressure ≥160/≥95 mmHg or both SBP ≥150 and DBP ≥90 mmHg, or antihypertensive treatment), CAD or meeting both criteria: history of >2 anginal attacks per week with stable frequency and ST-segment depression of ≥1 mm on stress test (or detection of MI with myocardial scintigraphy)	MI, unstable angina, DBP ≥120 mmHg, secondary hypertension, symptomatic CeVD, HF, atrial fibrillation/arrhythmias, renal or hepatic dysfunction, uncontrollable diabetes and familial hypercholesterolaemia	65 (85)	2.3	CCB (828)	ACEI (822)	372/1278	Diagnosis/history of diabetes mellitus	2.0	1.5
31.	LIFE[31]	Drug classes comparison	Age 55-80 years, hypertension (SBP 160-200 mmHg; DBP 95-115 mmHg), electrocardiogram signs of LVH	Secondary hypertension, recent MI or stroke, angina pectoris requiring treatment, HF or LEF ≤40%	67 (7)	4.9	ARB (4605)	Beta-blocker (4588)	1195/7998	Diagnosis/history of diabetes mellitus	1.2	0.5
32.	MOSES[32]	Drug classes comparison	Hypertension requiring treatment, documented TIA, ischaemic stroke or cerebral haemorrhage	Internal carotid artery occlusion or stenosis >70%, HF, age >85 years, on anticoagulant for cardiac arrhythmia, high-grade aortic or mitral valve stenosis, unstable angina	68 (10)	3.3	CCB (671)	ARB (681)	498/854	Diagnosis/history of diabetes mellitus	1.5	0.5
33.	NICS-EH[33]	Drug classes comparison	Age ≥60 years, SBP 160-220 mmHg and DBP <115 mmHg and no cardiovascular complications	None specified	70 (7)	3.2	Diuretic (214)	CCB (215)	17/412	Diagnosis/history of diabetes mellitus	0.3	0.7
34.	NORDIL[34]	Drug classes comparison	Age 50-74 years, untreated hypertension (DBP ≥100 mmHg on two occasions); if previously treated, DBP ≥100 mmHg on two consecutive visits at one week apart during run-in period and no treatment was given	Age <50 or ≥70y, bradycardia, secondary hypertension, atrial fibrillation, recent CeVD or MI, HF	60 (7)	4.2	Beta-blocker and/or Diuretic (5471)	CCB (5410)	727/10154	Diagnosis/history of type 2 (non-insulin dependent) diabetes mellitus	3.3	0.1
35.	ONTARGET[35]	Drug classes comparison	CAD, PAD, CeVD or diabetes with end-organ damage	HF, pericarditis, CHD, unexplained syncope, planned revascularisation <3 months of consent, uncontrolled hypertension, heart transplant, subarachnoid haemorrhage, renal artery disease, proteinuria, hepatic dysfunction, volume or	67 (7)	4.8	ARB/ACEI (8502)	ACEI and ARB (17118)	9612/16001	Diagnosis/history of type 2 diabetes mellitus with end-organ damage	1.9	1.0

				sodium depletion, primary hyper-aldosteronism, hereditary fructose intolerance, other serious conditions								
36.	PART 2[36]	Placebo-controlled	Age ≤75 years, diagnosis (in past 5 year) of MI, documented CAD, TIA or intermittent claudication	HF, serious nonvascular disease, SBP >160 mmHg, DBP >100 mm Hg, DBP <100 mmHg during pre-randomization run-in period	60 (8)	4.6	ACEI (308)	Placebo (309)	51/566	Diagnosis/history of diabetes mellitus	6.5	3.6
37.	PEACE[37]	Placebo-controlled	Age ≥50 years, documented CAD	Unstable angina, severe valvular heart disease, recent revascularisation, planned elective revascularisation, limited 5-year survival, serum creatinine >177 μmol/l, serum potassium >5.5 mmol/l	64 (8)	4.7	ACEI (4158)	Placebo (4132)	1380/6910	Diagnosis/history of diabetes mellitus	3.0	1.5
38.	PREVEND IT[38]	Placebo-controlled	Microalbuminuria, SBP <160/100 mmHg (no previous antihypertension treatment)	Creatinine clearance <60% of normal age-adjusted value	51 (12)	3.8	ACEI (431)	Placebo (433)	43/821	Diagnosis/history of diabetes mellitus, or fasting blood glucose ≥126 mg/dL [7 mmol/l]	5.6	2.8
39.	PREVENT[39]	Placebo-controlled	Age 30-80 years, documented CAD, DBP <95 mmHg, cholesterol <325 mg/dl, fasting blood glucose <200 mg/dl	Contraindication for dihydropyridines, uncontrolled hypertension, diabetes and other major illness	57 (10)	3.0	CCB (417)	Placebo (408)	98/727	Diagnosis/history of diabetes mellitus	6.1	3.3
40.	PROFESS[40]	Placebo-controlled	Age ≥55 years with ischaemic stroke <90 days before randomization (later modified to include age 50 to 54 years or had stroke 90 to 120 days before randomisation if with ≥2 additional risk factors: diabetes, hypertension, smoker, obesity previous CVD, end-organ damage or hyperlipidaemia) and remained stable[a]	Haemorrhagic stroke, severe disability after the qualifying stroke, contraindication to treatments	66 (8)	2.5	ARB (9873)	Placebo (9925)	5587/14211	Diagnosis/history of diabetes mellitus	3.4	3.4
41.	PROGRESS[41]	Placebo-controlled	Stroke or TIA in past 5 years	Indication or contraindication for ACEI	64 (10)	3.9	ACEI and/or Diuretic (3051)	Placebo (3054)	761/5344	Diagnosis/history of diabetes mellitus	9.2	4.0
42.	SHEP[42]	Placebo-controlled	Age ≥60 years, isolated systolic hypertension (BP 160-219/<90 mmHg, not on treatment)	Major CVD, cancer, alcoholic liver disease, renal dysfunction, competing risk of SHEP primary endpoint or presence of medical management exclusions	72 (7)	5.0	Beta-blocker and Diuretic (2365)	Placebo (2371)	476/4238	Diagnosis/history of type 2 diabetes mellitus	12.8	4.2
43.	SPRINT[43]	Intensive	Age ≥50 y years, SBP 130-180 mmHg, increased CVD risk (clinical/subclinical CVD other than stroke, CKD excluding polycystic kidney disease and with eGFR of 20-60	Prior stroke	68 (9)	3.0	More intensive (4678)	Less intensive (4683)	394/8967	Diagnosis/history of diabetes mellitus, fasting glucose at randomization ≥126 mg/dL (7 mmol/L), treatment with hypoglycaemic agents	14.9	7.7

			ml/min/1.73m^2 body surface area, 10-year Framingham CVD risk ≥15%, age ≥75y)									
44.	STOP Hypertension-2[44]	Drug classes comparison	Aged 70-84 years, SBP ≥180 mmHg and/or DBP ≥105 mmHg	Not specified	76 (4)	4.5	Beta-blocker and/or Diuretic (2213)	ACEI and CCB (4401)	719/5895	Diagnosis/history of diabetes mellitus	2.1	0.3
45.	SYST-EUR[45]	Placebo-controlled	Age ≥60 years, sitting SBP 160-219 mmHg, sitting DBP <95 mmHg, and standing SBP ≥140 mmHg	Secondary hypertension, retinal haemorrhage/papilloedema, HF, dissecting aortic aneurysm, serum creatinine ≥180 μmol/l, recent severe nosebleeds, stroke or MI, dementia, disorders prohibiting standing position, severe CVD/non-CVD	70 (7)	2.6	CCB (2398)	Placebo (2297)	584/4111	Diagnosis/history of diabetes mellitus, or fasting glucose at randomization ≥ 126 mg/dL (7 mmol/L)	10.1	4.0
46.	TRANSCEND[46]	Placebo-controlled	Intolerant to ACEI and with established CAD, PVD, CeVD or diabetes with end-organ damage	HF, valvular/cardiac outflow tract obstruction, pericarditis, congenital heart disease, unexplained syncope, recent revascularisation, SBP >160 mmHg, heart transplantation, subarachnoid haemorrhage, significant renal stenosis, renal or hepatic dysfunction	68 (7)	4.9	ARB (2954)	Placebo (2972)	2284/3642	Diagnosis/history of diabetes mellitus, or fasting glucose at randomization ≥ 126 mg/dL (7 mmol/l)	4.5	2.2
47.	UKPDS[47]	Intensive	Age 25-65 years, newly-diagnosed diabetes, and hypertension (untreated: SBP ≥160 mmHg and/or DBP ≥90 mmHg; treated: SBP ≥150 mmHg and/or DBP ≥85 mmHg)	Ketonuria, recent MI, angina, HF, >1 major vascular episode, serum creatinine >15 μmol/, retinopathy, malignant hypertension, uncorrected endocrine abnormality, severe concurrent illness	56 (8)	7.9	More intensive (758)	Less intensive (390)	1148/0	Fasting plasma glucose concentration > 108 mg/dl (6 mmol/l) on two mornings	11.2	1.2
48.	VALISH[48]	Intensive	Age ≥70 to <85 years, isolated hypertension (SBP >160 mmHg and DBP <90 mmHg)	Secondary or malignant hypertension, BP ≥200/≥90 mmHg, recent CeVD or MI, recent/planned revascularisation, HF, aortic stenosis, valvular heart disease, atrial fibrillation/flutter, serious arrhythmia, renal/liver dysfunction	76 (4)	2.6	More intensive (1545)	Less intensive (1534)	418/2661	Diagnosis/history of diabetes mellitus, or fasting glucose at randomization ≥126 mg/dL (7 mmol/L)	5.0	1.8
49.	VALUE[49]	Drug classes comparison	Age ≥50 years, hypertension, CVD, CVD risk factors (male sex, age >50 years, diabetes, current smoking, high cholesterol, LVH, proteinuria, serum creatinine 150 to 265 μmol/l)	Renal artery stenosis, recent CAD or CeVD, severe hepatic disease or chronic renal failure, HF, on monotherapy with beta-blocker for CAD and hypertension	67 (8)	4.2	CCB-based (7596)	ARB-based (7649)	5376/9869	Diagnosis/history of diabetes mellitus, or fasting glucose at randomization ≥126 mg/dl (7 mmol/L)	1.6	1.3
50.	VHAS[50]	Drug classes comparison	Age 40-65 years, BP ≥160/95 mmHg	Secondary hypertension, recent stroke or TIA, CAD, PAD, bradycardia, arrhythmias, HF, renal or hepatic dysfunction,	54 (7)	1.7	Diuretic (707)	CCB (707)	135/1279	Diagnosis/history of diabetes mellitus	1.7	1.3

				hyperuricaemia, hypokalemia, type 1 diabetes mellitus, familial dyslipidemia, serious concomitant disease								
51.	HDFP [51]	Intensive	Ages 30-69 years, hypertension, DBP home readings and clinic readings ≥ 95 mmHg and 90 mmHg, respectively	Bedfast or institutionalized persons	50.8	7.2	More intensive (5553)	Less intensive (5387)	894/10034	Diagnosis/history of diabetes mellitus, or random blood glucose > 200 mg/dl (11.1 mmol/L)	9.9	4.9

* Estimated through a one-stage individual patient data meta-analysis approach, and applied linear mixed models to estimate the effect of treatment on blood pressure between comparison arms, SD: standard deviation, SBP: systolic blood pressure, DBP: diastolic blood pressure, eGFR: estimated glomerular filtration rate, CVD: cardiovascular diseases, LVH: left ventricular hypertrophy, TIA: transient ischaemic attack, CNS: central nervous system, LVEF: left ventricular ejection fraction, PVD: peripheral vascular disease, CAD: coronary artery disease, MI: myocardial infarction, HDL: high-density lipoprotein, ECG: electrocardiography, TC: total cholesterol, CeVD: cerebrovascular disease, CHD: coronary artery disease, CKD: coronary artery disease, ACEI: Angiotensin-converting enzyme inhibitors, ARB: angiotensin receptor blockers, CCB: calcium channel blocker

AASK: African American Study of Kidney Disease and Hypertension; ABCD: Appropriate Blood Pressure Control in Diabetes, ACCORD: Action to Control Cardiovascular Risk in Diabetes blood pressure trial; ACTIVE I: Atrial Fibrillation Clopidogrel Trial with Irbesartan for Prevention of Vascular Events, ADVANCE: Action in Diabetes and Vascular disease: preterAx and diamicroN-MR Controlled Evaluation, ALLHAT: Antihypertensive and Lipid Lowering Treatment to Prevent Heart Attack Trial, ANBP: Australian National Blood Pressure Study, ANBP2: Second Australian National Blood Pressure Study, ASCOT-BPLA: Anglo-Scandinavian Cardiac Outcomes Trial-Blood Pressure Lowering Arm, BENEDICT: BErgamo NEphrologic DIabetes Complications Trial, CAMELOT: Comparison of Amlodipine vs Enalapril to Limit Occurrences of Thrombosis, CAPPP: Captopril Prevention Project, Cardio-Sis: Studio Italiano Sugli Effetti Cardiovascolari del Controllo della Pressione Arteriosa Sistolica, CASE-J: Candesartan Antihypertensive Survival Evaluation in Japan Trial, COLM: Combination of OLMesartan study, CONVINCE: Controlled ONset Verapamil INvestigation of Cardiovascular Endpoints trial, COPE: Combination Therapy of Hypertension to Prevent Cardiovascular Events, DIABHYCAR: Noninsulin-dependent diabetes, hypertension, microalbuminuria or proteinuria, cardiovascular events, and ramipril, Dutch TIA Trial: Dutch Transient Ischemic Attack Trial, E-COST: Efficacy of Candesartan on Outcome in Saitama Trial, ELSA: European Lacidipine Study on Atherosclerosis, EUROPA: EUropean trial on Reduction Of cardiac events with Perindopril in patients with stable coronary Artery disease, EWPHE: European Working Party on High Blood Pressure in the Elderly, HIJ-CREATE: Heart Institute of Japan Candesartan Randomized Trial for Evaluation in Coronary Artery Disease, HOMED-BP: Hypertension Objective Treatment Based on Measurement by Electrical Devices of Blood Pressure, HOPE: Heart Outcomes Prevention Evaluation, HYVET: Hypertension in the Very Elderly Trial, IDNT: Irbesartan Diabetic Nephropathy Trial, INSIGHT: International Nifedipine GITS study: Intervention as a Goal in Hypertension Treatment, INVEST: International Verapamil-Trandolapril Study, JMIC-B: Japan Multicenter Investigation for Cardiovascular Diseases-B, LIFE: Losartan Intervention For Endpoint reduction, MOSES: Morbidity and Mortality After Stroke, Eprosartan Compared With Nitrendipine for Secondary Prevention, NICS-EH: National Intervention Cooperative Study in Elderly Hypertensives, NORDIL: Nordic Diltiazem Study, ONTARGET: Ongoing Telmisartan Alone and in Combination with Ramipril Global Endpoint Trial, PART 2: Prevention of Atherosclerosis with Ramipril Trial, PEACE: Prevention of Events with Angiotensin Converting Enzyme Inhibition, PREVEND IT: Prevention of Renal and Vascular Endstage Disease Intervention Trial, PREVENT: Prospective Randomized Evaluation of the Vascular Effects of Norvasc Trial, PROFESS: Prevention Regimen for Effectively Avoiding Second Strokes, PROGRESS: Perindopril Protection Against Recurrent Stroke Study, SHEP: Systolic Hypertension in the Elderly Program, SPRINT: Systolic Blood Pressure Intervention Trial, STOP: Hypertension-2 Swedish Trial in Old Patients with Hypertension-2, Syst-Eur: Systolic Hypertension in Europe, TRANSCEND: Telmisartan Randomized Assessment Study in ACE Intolerant Subjects with Cardiovascular Disease, UKPDS: UK Prospective Diabetes Study, VALISH: Valsartan in Elderly Isolated Systolic Hypertension, VALUE: Valsartan Antihypertensive Long-term Use Evaluation, VHAS: Verapamil in Hypertension and Atherosclerosis Study

Table S2. Sensitivity analysis excluding head-to-head trials for the effect of blood pressure-lowering treatment on primary and secondary outcomes, by type 2 diabetes status at baseline.

Diabetes status	Intervention		Comparator					
	Events	Total	Events	Total	Hazard ratio	95% confidence interval		Adjusted P for interaction
**Major cardiovascular events**								
Previous diabetes	3639	22787	3821	21722	0.94	0.89	0.98	
No previous diabetes	5016	49827	5871	49066	0.86	0.83	0.9	
Overall	8655	72614	9692	70788	0.89	0.87	0.92	0.003
**Stroke**								
Previous diabetes	1139	22805	1305	21744	0.87	0.80	0.94	
No previous diabetes	2076	49896	2474	49133	0.86	0.80	0.92	
Overall	3215	72701	3779	70877	0.88	0.83	0.91	1.00
**Ischaemic heart disease**								
Previous diabetes	1376	22785	1434	21720	0.90	0.83	0.97	
No previous diabetes	2111	49837	2433	49075	0.85	0.79	0.91	
Overall	3487	72622	3867	70795	0.87	0.82	0.91	0.28
**Heart failure**								
Previous diabetes	680	19966	732	19301	0.93	0.84	1.03	
No previous diabetes	659	44281	808	43459	0.84	0.75	0.94	
Overall	1339	64247	1540	62760	0.88	0.82	0.95	0.10
**Cardiovascular death**								
Previous diabetes	948	19541	925	18826	1.05	0.96	1.15	
No previous diabetes	1368	41155	1556	40406	0.93	0.86	1.02	
Overall	2316	60696	2481	59232	0.98	0.92	1.04	0.007
**All-cause death**								
Previous diabetes	2447	23975	2428	22335	0.95	0.90	1	
No previous diabetes	3650	49905	3944	49129	0.95	0.90	1	
Overall	6097	73880	6372	71464	0.95	0.91	0.98	1.00

Hazard ratios standardised for blood pressure reduction across trials and rescaled to a fixed amount of 5 mmHg reduction in systolic blood pressure.

Figure S1. Meta-regression of intensity of blood pressure reduction and hazard ratio of major cardiovascular events, by type 2 diabetes status at baseline.

The hazard ratio for each trial is shown by the centre of the bubbles, with the size of the bubble inversely related to the respective standard error. The solid red line is the fitted regression line; the dotted blue lines represent the 95 per cent confidence intervals; the dashed grey line represents hazard ratio=1. Regression coefficients and 95 per cent confidence intervals in people with and without diabetes at baseline were - 0.007 (-0.036 to 0.0205) and -0.014 (-0.035 to 0.006), respectively.

Diabetes

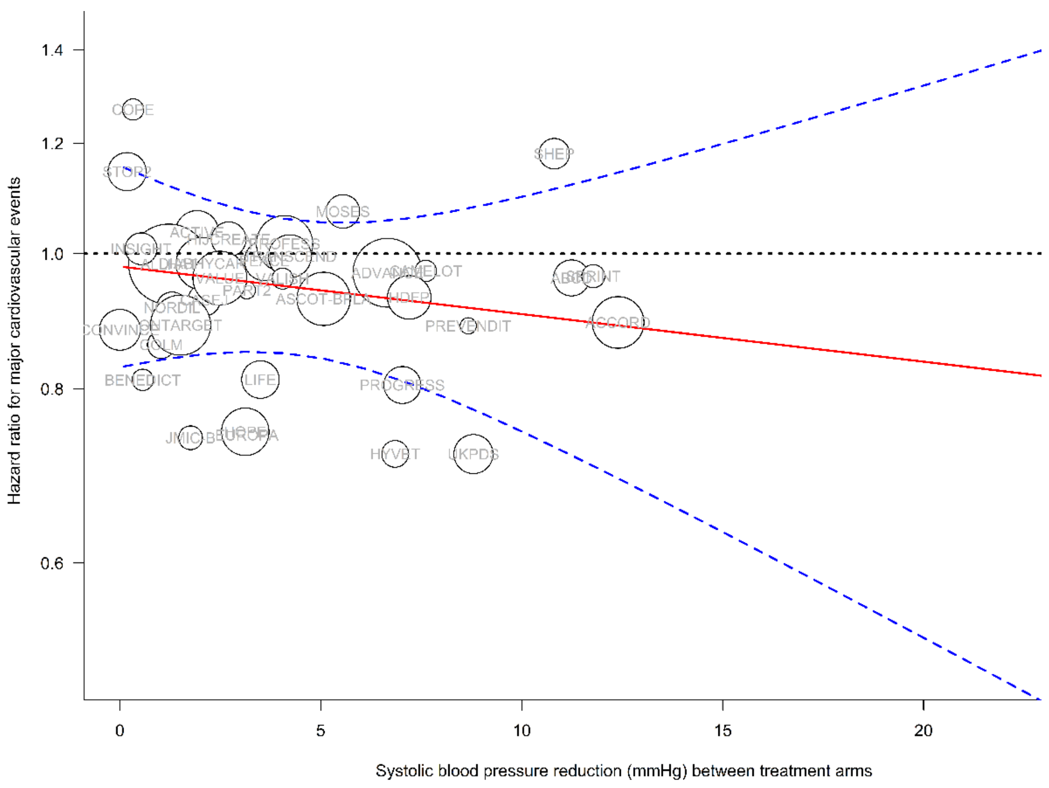

No diabetes

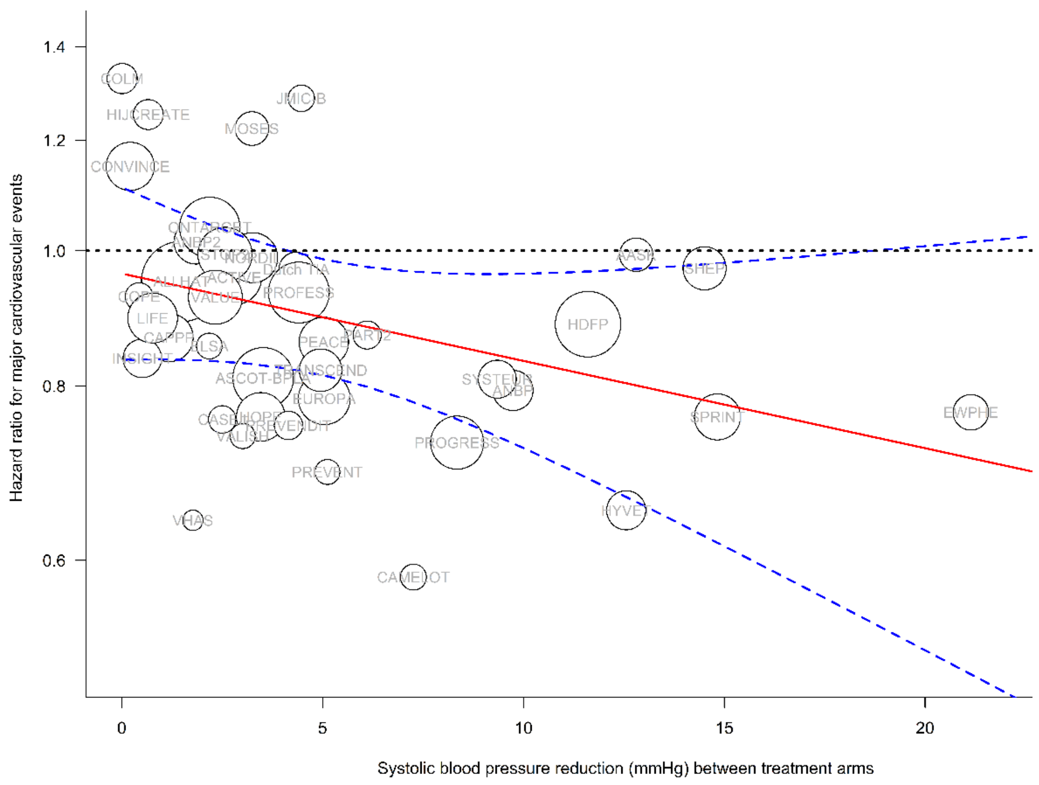

Figure S2. Effects of blood pressure-lowering treatment on primary and secondary outcomes stratified by baseline systolic blood pressure and type 2 diabetes at baseline.

Hazard ratios standardised for blood pressure reduction across trials and rescaled to a fixed amount of 5 mmHg reduction in systolic blood pressure.

Previous diabetes at baseline

	Intervention Events	Intervention Total	Comparator Events	Comparator Total	Hazard ratio per 5 mmHg reduction in systolic blood pressure	95% CI
**Major cardiovascular events**						
<120 mmHg	362	2427	464	2683	0.87	[0.76; 1.00]
120-129 mmHg	646	4183	836	4981	0.96	[0.86; 1.07]
130-139 mmHg	1094	7199	1347	8423	1.00	[0.92; 1.09]
140-149 mmHg	1525	9862	1916	11544	0.95	[0.88; 1.03]
150-159 mmHg	1335	8641	1786	10245	0.89	[0.82; 0.97]
160-169 mmHg	1060	7233	1345	7894	0.88	[0.80; 0.96]
≥170 mmHg	1412	7571	1632	8160	0.95	[0.88; 1.03]
Adjusted p interaction 1.00						
**Stroke**						
<120 mmHg	82	2429	102	2684	0.91	[0.66; 1.25]
120-129 mmHg	157	4183	200	4981	0.93	[0.73; 1.18]
130-139 mmHg	315	7204	365	8424	0.94	[0.79; 1.11]
140-149 mmHg	449	9862	555	11548	0.86	[0.73; 1.01]
150-159 mmHg	375	8645	532	10253	0.79	[0.67; 0.93]
160-169 mmHg	347	7241	481	7911	0.78	[0.67; 0.90]
≥170 mmHg	511	7583	636	8177	0.86	[0.76; 0.98]
Adjusted p interaction 1.00						
**Ischaemic heart disease**						
<120 mmHg	174	2425	224	2683	0.91	[0.74; 1.11]
120-129 mmHg	310	4183	428	4980	0.93	[0.80; 1.08]
130-139 mmHg	507	7198	672	8423	1.00	[0.89; 1.12]
140-149 mmHg	719	9857	942	11538	1.00	[0.90; 1.11]
150-159 mmHg	670	8637	913	10239	0.91	[0.82; 1.01]
160-169 mmHg	502	7240	634	7909	0.90	[0.79; 1.02]
≥170 mmHg	672	7583	773	8175	1.02	[0.92; 1.14]
Adjusted p interaction 1.00						
**Heart failure**						
<120 mmHg	90	2254	126	2500	0.84	[0.63; 1.11]
120-129 mmHg	167	3891	206	4743	1.04	[0.84; 1.29]
130-139 mmHg	223	6728	346	8051	0.89	[0.74; 1.08]
140-149 mmHg	370	9344	473	11077	1.00	[0.84; 1.19]
150-159 mmHg	304	8141	475	9765	0.84	[0.70; 1.01]
160-169 mmHg	233	6515	347	7308	0.80	[0.63; 1.01]
≥170 mmHg	277	6788	340	7403	0.93	[0.78; 1.11]
Adjusted p interaction 1.00						
**Cardiovascular death**						
<120 mmHg	108	2201	116	2457	1.12	[0.83; 1.52]
120-129 mmHg	145	3775	204	4620	0.95	[0.75; 1.21]
130-139 mmHg	277	6588	344	7889	1.00	[0.84; 1.20]
140-149 mmHg	410	9222	481	10954	1.00	[0.85; 1.18]
150-159 mmHg	385	8132	449	9747	1.06	[0.89; 1.27]
160-169 mmHg	307	6759	380	7538	1.01	[0.86; 1.19]
≥170 mmHg	450	7221	492	7878	1.07	[0.94; 1.22]
Adjusted p interaction 1.00						
**All-cause death**						
<120 mmHg	251	2428	308	2690	0.92	[0.76; 1.11]
120-129 mmHg	418	4187	500	4984	1.10	[0.95; 1.28]
130-139 mmHg	728	7213	930	8431	0.98	[0.88; 1.09]
140-149 mmHg	1047	9868	1217	11554	1.01	[0.91; 1.13]
150-159 mmHg	955	8652	1193	10256	0.94	[0.84; 1.05]
160-169 mmHg	775	7244	946	7918	0.96	[0.86; 1.07]
≥170 mmHg	1036	7588	1135	8176	1.01	[0.92; 1.10]
Adjusted p interaction 1.00						

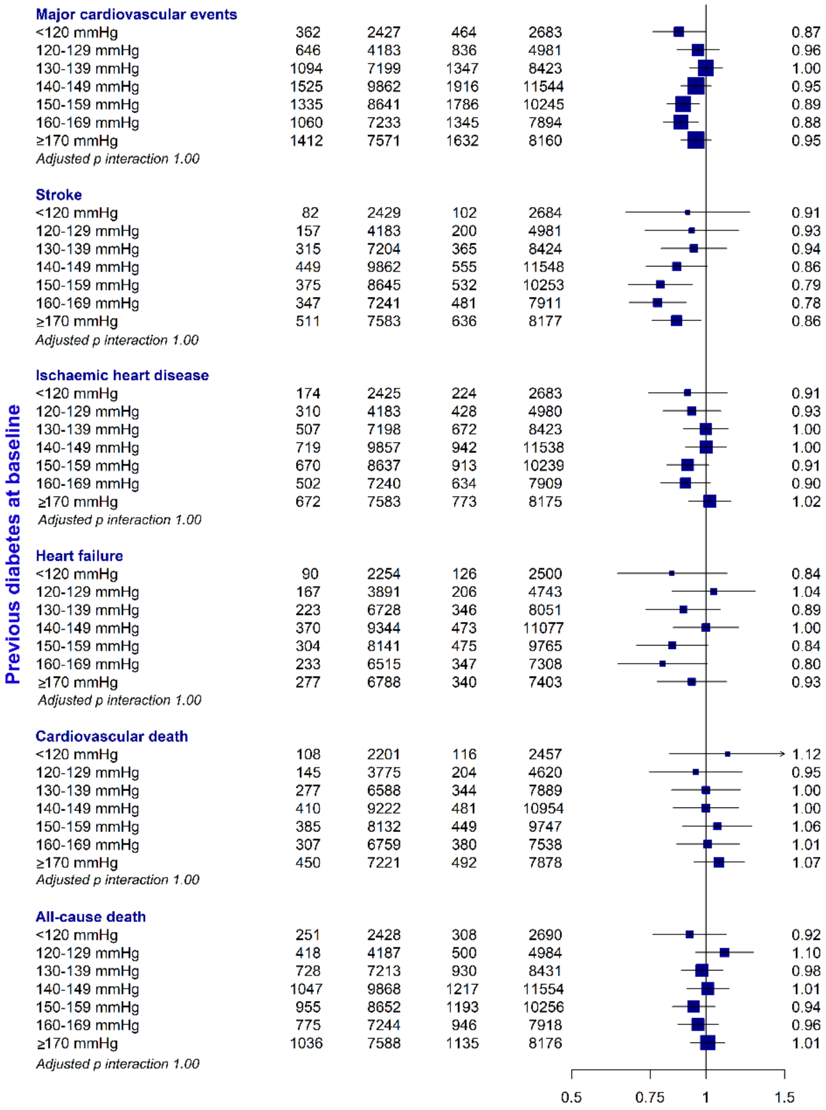

No previous diabetes at baseline

	Intervention Events	Intervention Total	Comparator Events	Comparator Total	Hazard ratio per 5 mmHg reduction in systolic blood pressure	95% CI
**Major cardiovascular events**						
<120 mmHg	467	5361	707	6192	0.75	[0.66; 0.86]
120-129 mmHg	912	9739	1203	11146	0.88	[0.80; 0.96]
130-139 mmHg	1481	15136	1864	17178	0.92	[0.86; 0.99]
140-149 mmHg	1910	20862	2536	23580	0.89	[0.83; 0.95]
150-159 mmHg	1826	19658	2438	22089	0.90	[0.84; 0.96]
160-169 mmHg	1827	22193	2270	23258	0.88	[0.83; 0.94]
≥170 mmHg	2943	27540	3764	30088	0.89	[0.85; 0.94]
Adjusted p interaction 1.00						
**Stroke**						
<120 mmHg	115	5364	189	6198	0.73	[0.57; 0.94]
120-129 mmHg	277	9748	366	11151	0.85	[0.72; 1.00]
130-139 mmHg	493	15149	613	17187	0.91	[0.80; 1.03]
140-149 mmHg	667	20877	855	23590	0.86	[0.77; 0.96]
150-159 mmHg	634	19672	899	22109	0.81	[0.72; 0.91]
160-169 mmHg	742	22225	862	23291	0.91	[0.82; 1.00]
≥170 mmHg	1298	27597	1644	30134	0.91	[0.85; 0.98]
Adjusted p interaction 1.00						
**Ischaemic heart disease**						
<120 mmHg	237	5361	361	6189	0.81	[0.67; 0.98]
120-129 mmHg	464	9741	598	11141	0.95	[0.83; 1.08]
130-139 mmHg	736	15132	962	17178	0.93	[0.84; 1.03]
140-149 mmHg	955	20865	1344	23582	0.89	[0.81; 0.97]
150-159 mmHg	969	19663	1260	22093	0.96	[0.88; 1.05]
160-169 mmHg	852	22212	1107	23281	0.87	[0.79; 0.95]
≥170 mmHg	1350	27592	1750	30126	0.89	[0.83; 0.96]
Adjusted p interaction 1.00						
**Heart failure**						
<120 mmHg	106	4889	163	5693	0.71	[0.53; 0.95]
120-129 mmHg	175	8974	227	10455	0.91	[0.73; 1.14]
130-139 mmHg	242	14042	346	16116	0.80	[0.67; 0.96]
140-149 mmHg	298	19121	444	21964	0.88	[0.75; 1.04]
150-159 mmHg	285	17571	402	20117	0.92	[0.77; 1.09]
160-169 mmHg	219	18410	357	19378	0.65	[0.54; 0.79]
≥170 mmHg	294	21675	368	24031	0.89	[0.76; 1.04]
Adjusted p interaction 1.00						
**Cardiovascular death**						
<120 mmHg	118	4303	161	5209	0.71	[0.51; 0.99]
120-129 mmHg	186	8046	293	9415	0.77	[0.63; 0.95]
130-139 mmHg	371	13301	427	15296	0.98	[0.84; 1.14]
140-149 mmHg	465	19241	599	21950	0.88	[0.77; 1.01]
150-159 mmHg	452	18304	571	20727	0.97	[0.85; 1.11]
160-169 mmHg	499	21500	639	22604	0.87	[0.77; 0.98]
≥170 mmHg	1018	27205	1371	29742	0.88	[0.81; 0.96]
Adjusted p interaction 0.17						
**All-cause death**						
<120 mmHg	399	5366	518	6196	0.85	[0.73; 0.99]
120-129 mmHg	653	9747	853	11150	0.92	[0.82; 1.03]
130-139 mmHg	1077	15155	1262	17188	0.99	[0.91; 1.08]
140-149 mmHg	1399	20884	1716	23594	0.95	[0.88; 1.03]
150-159 mmHg	1338	19672	1652	22105	0.95	[0.87; 1.03]
160-169 mmHg	1341	22224	1600	23291	0.93	[0.87; 1.00]
≥170 mmHg	2311	27597	2821	30134	0.95	[0.90; 1.01]
Adjusted p interaction 1.00						

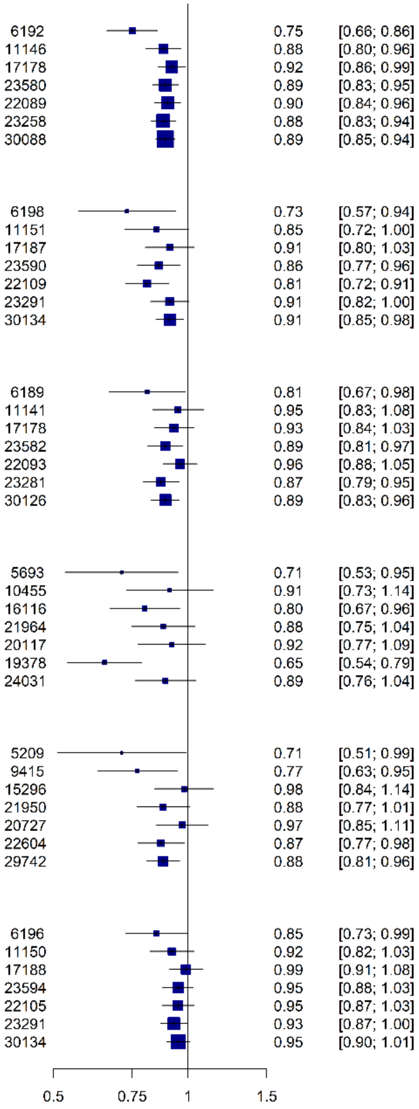

Figure S3. Effect of major antihypertensive drug classes on the risk major cardiovascular outcomes, by type 2 diabetes at baseline.

The relative risk for each trial was estimated using the binary logistic regression model. p for interaction calculated using Chi-square test for heterogeneity of effect and adjusted for multiple comparisons.

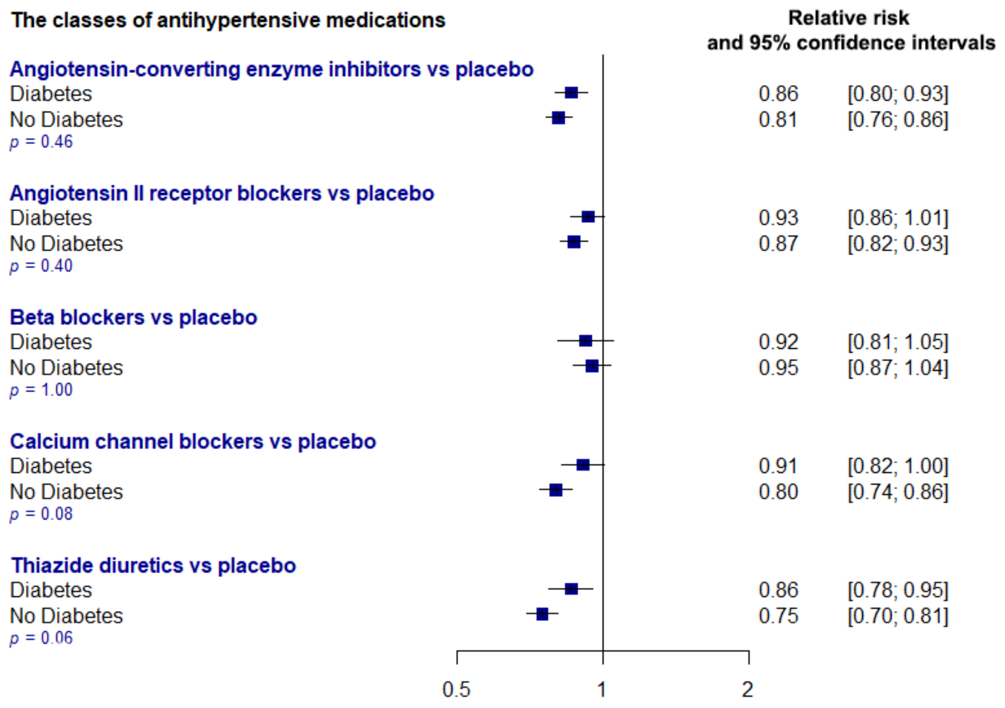

Figure S4. The unstandardised effects of blood pressure-lowering treatment on primary and secondary outcomes, by type 2 diabetes status at baseline.

	Intervention		Comparator		Hazard Ratio and 95% CIs	
	Events	Total	Events	Total		
**Major cardiovascular events**						
Previous diabetes	7441	47151	9335	53981	0.95	[0.92; 0.98]
No previous diabetes	11370	120551	14785	133595	0.90	[0.88; 0.93]
**Overall**	**18811**	**167702**	**24120**	**187576**	**0.92**	**[0.90; 0.94]**
Adjusted p interaction 0.001						
**Stroke**						
Previous diabetes	2238	47183	2872	54029	0.91	[0.86; 0.96]
No previous diabetes	4229	120695	5429	133725	0.88	[0.85; 0.92]
**Overall**	**6467**	**167878**	**8301**	**187754**	**0.89**	**[0.86; 0.92]**
Adjusted p interaction 0.68						
**Ischaemic heart disease**						
Previous diabetes	3557	47159	4590	53997	0.97	[0.93; 1.01]
No previous diabetes	5563	120629	7383	133655	0.92	[0.89; 0.95]
**Overall**	**9120**	**167788**	**11973**	**187652**	**0.94**	**[0.91; 0.96]**
Adjusted p interaction 0.01						
**Heart failure**						
Previous diabetes	1666	43685	2314	50886	0.91	[0.86; 0.97]
No previous diabetes	1620	104734	2308	117809	0.87	[0.82; 0.93]
**Overall**	**3286**	**148419**	**4622**	**168695**	**0.89**	**[0.85; 0.93]**
Adjusted p interaction 0.38						
**Cardiovascular death**						
Previous diabetes	2084	43924	2466	51069	1.05	[0.99; 1.11]
No previous diabetes	3109	111963	4062	125007	0.91	[0.87; 0.96]
**Overall**	**5193**	**155887**	**6532**	**176129**	**0.96**	**[0.93; 1.00]**
Adjusted p interaction <0.001						
**All-cause death**						
Previous diabetes	5379	48357	6331	54627	1.00	[0.96; 1.04]
No previous diabetes	8521	120708	10427	133723	0.97	[0.94; 1.00]
**Overall**	**13900**	**169065**	**16758**	**188350**	**0.98**	**[0.96; 1.00]**
Adjusted p interaction 0.06						

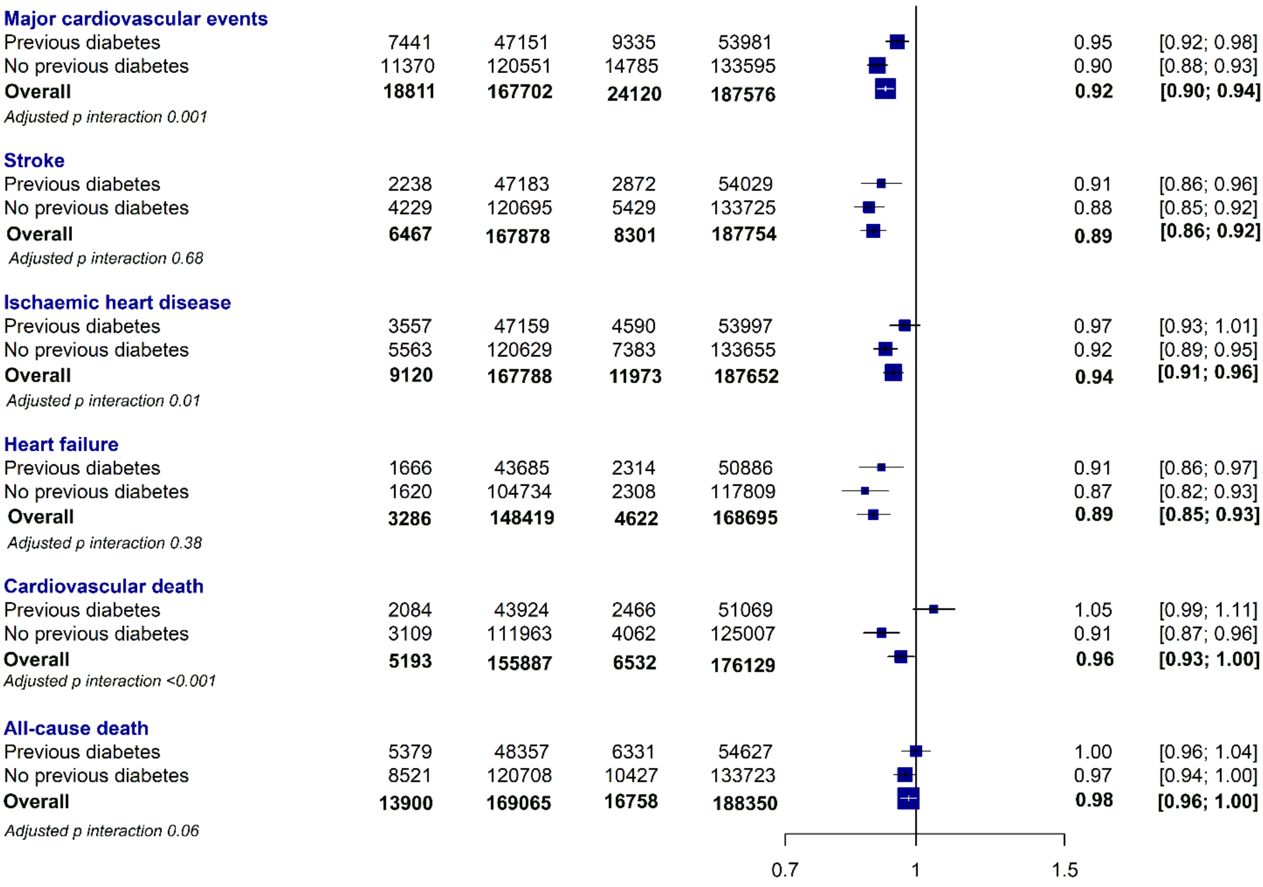

Figure S5. Sensitivity analysis restricted to trials that used a laboratory test for diagnosis of type 2 diabetes at baseline.

	Intervention Events	Intervention Total	Comparator Events	Comparator Total	Hazard ratio and 95% CIs per 5 mmHg reduction in systolic blood pressure	
**Major cardiovascular events**						
Previous diabetes	3474	19986	4638	24135	0.95	[0.91; 0.99]
No previous diabetes	5500	52661	7398	59124	0.88	[0.85; 0.91]
Overall	8974	72647	12036	83259	0.91	[0.89; 0.94]
Adjusted p interaction 0.003						
**Stroke**						
Previous diabetes	854	19982	1216	24128	0.81	[0.74; 0.89]
No previous diabetes	1702	52676	2230	59134	0.85	[0.80; 0.90]
Overall	2556	72658	3446	83262	0.84	[0.80; 0.89]
Adjusted p interaction 1.00						
**Ischaemic heart disease**						
Previous diabetes	2243	19984	2963	24133	1.00	[0.95; 1.06]
No previous diabetes	3247	52662	4409	59124	0.92	[0.88; 0.96]
Overall	5490	72646	7372	83257	0.95	[0.92; 0.98]
Adjusted p interaction 0.01						
**Heart failure**						
Previous diabetes	895	18917	1259	23376	0.93	[0.85; 1.02]
No previous diabetes	841	46788	1312	53291	0.79	[0.72; 0.86]
Overall	1736	65705	2571	76667	0.85	[0.80; 0.90]
Adjusted p interaction 0.02						
**Cardiovascular death**						
Previous diabetes	907	19229	1192	23745	1.03	[0.94; 1.13]
No previous diabetes	1491	52142	2042	58604	0.88	[0.82; 0.94]
Overall	2398	71371	3234	82349	0.93	[0.88; 0.98]
Adjusted p interaction 0.001						
**All-cause death**						
Previous diabetes	2478	19993	2906	24140	1.05	[0.99; 1.11]
No previous diabetes	4014	52681	4948	59139	0.95	[0.91; 0.99]
Overall	6492	72674	7854	83279	0.98	[0.95; 1.01]
Adjusted p interaction 0.001						

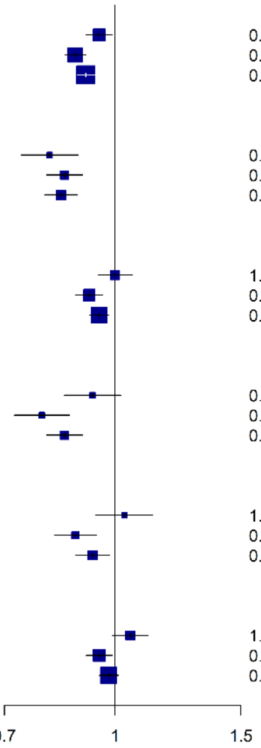

References

1. Appel LJ, Wright JT, Greene T. Intensive blood-pressure control in hypertensive chronic kidney disease. New England Journal of Medicine 2010;363(26):2565–6.

2. Schrier RW, Estacio RO, Jeffers B. Appropriate Blood Pressure Control in NIDDM (ABCD) Trial. Diabetologia [Internet] 1996;39(12):1646–54.

3. Cushman WC, Evans GW, Byington RP, et al. Effects of intensive blood-pressure control in type 2 diabetes mellitus. New England Journal of Medicine 2010;362(17):1575–85.

4. Yusuf S, Healey JS, Pogue J, et al. Irbesartan in patients with atrial fibrillation. New England Journal of Medicine 2011;364(10):928–38.

5. Patel A. Effects of a fixed combination of perindopril and indapamide on macrovascular and microvascular outcomes in patients with type 2 diabetes mellitus (the ADVANCE trial): a randomised controlled trial. Lancet 2007;370(9590):829–40.

6. Group TAO and C for the ACR, Coordinators TAO and, Antihypertensive T, Treatment L. Major Outcomes in High-Risk Hypertensive Patients Randomized to Angiotensin-Converting Enzyme Inhibitor or Calcium Channel Blocker vs Diuretic. JAMA: The Journal of the American Medical Association 2002;288(23):2981–97.

7. Doyle AE. The Australian National blood pressure study. Trends in Pharmacological Sciences 1981;2(C):293–6.

8. Wing LMH, Reid CM, Ryan P, et al. A comparison of outcomes with angiotensin-converting-enzyme inhibitors and diuretics for hypertension in the elderly. New England Journal of Medicine 2003;348(7):583–92.

9. Dahlöf B, Sever PS, Poulter NR, et al. Prevention of cardiovascular events with an antihypertensive regimen of amlodipine adding perindopril as required versus atenolol adding bendroflumethiazide as required, in the Anglo-Scandinavian Cardiac Outcomes Trial-Blood Pressure Lowering Arm (ASCOT-B. Lancet 2005;366(9489):895–906.

10. Ruggenenti P, Fassi A, Ilieva AP, et al. Preventing microalbuminuria in type 2 diabetes. New England Journal of Medicine 2004;351(19):1941–51.

11. Park S, Yan P, Cerezo C, Jeffers BW. Effect of visit-to-visit blood pressure variability on cardiovascular events in patients with coronary artery disease and well-controlled blood pressure. Journal of the American Society of Hypertension 2016;10(10):799–810.

12. Hansson L, Lindholm LH, Niskanen L, et al. Effect of angiotensin-converting-enzyme inhibition compared with conventional therapy on cardiovascular morbidity and mortality in hypertension: the Captopril Prevention Project (CAPPP) randomised trial. Lancet 1999;353(9153):611–6.

13. Verdecchia P, Staessen JA, Angeli F, et al. Usual versus tight control of systolic blood pressure in non-diabetic patients with hypertension (Cardio-Sis): an open-label randomised trial. The Lancet 2009;374(9689):525–33.

14. Nakao K, Hirata M, Oba K, et al. Role of diabetes and obesity in outcomes of the candesartan antihypertensive survival evaluation in Japan (CASE-J) trial. Hypertension Research 2010;33(6):600–6.

15. Ogihara T, Saruta T, Rakugi H, et al. Combinations of olmesartan and a calciumchannel blocker or a diuretic inelderly hypertensive patients: A randomized, controlled trial. Journal of Hypertension 2014;32(10):2054–63.

16. Black HR, Elliott WJ, Grandits G, et al. Principal Results of the Controlled Onset Verapamil Investigation of Cardiovascular End Points (CONVINCE) Trial. Journal of the American Medical Association 2003;289(16):2073–82.

17. Matsuzaki M, Ogihara T, Umemoto S, et al. Prevention of cardiovascular events with calcium channel

blocker-based combination therapies in patients with hypertension: A randomized controlled trial. Journal of Hypertension 2011;29(8):1649–59.

18. Marre M, Lievre M, Chatellier G, Mann JFE, Passa P, Ménard J. Effects of low dose ramipril on cardiovascular and renal outcomes in patients with type 2 diabetes and raised excretion of urinary albumin: Randomised, double blind, placebo controlled trial (the DIABHYCAR study). British Medical Journal 2004;328(7438):495–9.

19. Koudstaal PJ, Algra A, Pop GA, Kappelle LJ, van Latum JC, van Gijn J. Risk of cardiac events in atypical transient ischaemic attack or minor stroke. The Dutch TIA Study Group. Lancet (London, England) 1992;340(8820):630–3.

20. Zanchetti A, Bond MG, Hennig M, et al. Calcium antagonist lacidipine slows down progression of asymptomatic carotid atherosclerosis: Principal results of the European Lacidipine Study on Atherosclerosis (ELSA), a randomized, double-blind, long-term trial. Circulation 2002;106(19):2422–7.

21. Fox KM, Bertrand M, Ferrari R, et al. Efficacy of perindopril in reduction of cardiovascular events among patients with stable coronary artery disease: Randomised, double-blind, placebo-controlled, multicentre trial (the EUROPA study). Lancet 2003;362(9386):782–8.

22. Amery A, Brixko P, Clement D, et al. Mortality and morbidity results from the European Working Party on High Blood Pressure in the Elderly trial. The Lancet 1985;325(8442):1349–54.

23. Kasanuki H, Hagiwara N, Hosoda S, et al. Angiotensin II receptor blocker-based vs. non-angiotensin II receptor blocker-based therapy in patients with angiographically documented coronary artery disease and hypertension: The Heart Institute of Japan Candesartan Randomized Trial for Evaluation in. European Heart Journal 2009;30(10):1203–12.

24. Asayama K, Ohkubo T, Metoki H, et al. Cardiovascular outcomes in the first trial of antihypertensive therapy guided by self-measured home blood pressure. Hypertension Research. 2012;35(11):1102–10.

25. Sharma AM, Pischon T, Engeli S. Effect of ramipril on cardiovascular events in high-risk patients. New England Journal of Medicine 2000;343(1).

26. Beckett NS, Peters R, Fletcher AE, et al. Treatment of hypertension in patients 80 years of age or older. New England Journal of Medicine 2008;358(18):1887–98.

27. Lewis EJ, Hunsicker LG, Clarke WR, et al. Renoprotective effect of the angiotensin-receptor antagonist irbesartan in patients with nephropathy due to type 2 diabetes. New England Journal of Medicine 2001;345(12):851–60.

28. Brown MJ, Palmer CR, Castaigne A, et al. Morbidity and mortality in patients randomised to double-blind treatment with a long-acting calcium-channel blocker or diuretic in the International Nifedipine GITS study: Intervention as a Goal in Hypertension Treatment (INSIGHT). Lancet 2000;356(9227):366–72.

29. Pepine CJ, Handberg EM, Cooper-DeHoff RM, et al. A Calcium Antagonist vs a Non-Calcium Antagonist Hypertension Treatment Strategy for Patients with Coronary Artery Disease the International Verapamil-Trandolapril Study (INVEST): A Randomized Controlled Trial. Journal of the American Medical Association 2003;290(21):2805–16.

30. Yui Y, Sumiyoshi T, Kodama K, et al. Comparison of nifedipine retard with angiotensin converting enzyme inhibitors in Japanese hypertensive patients with coronary artery disease: The Japan Multicenter Investigation for Cardiovascular Diseases-B (JMIC-B) randomized trial. Hypertension Research 2004;27(3):181–91.

31. Lindholm LH, Ibsen H, Dahlöf B, et al. Cardiovascular morbidity and mortality in patients with diabetes in the Losartan Intervention For Endpoint reduction in hypertension study (LIFE): A randomised trial against atenolol. Lancet 2002;359(9311):1004–10.

32. Schrader J, Lüders S, Kulschewski A, et al. Morbidity and mortality after stroke, eprosartan compared with nitrendipine for secondary prevention: Principal results of a prospective randomized controlled study (MOSES). Stroke 2005;36(6):1218–24.

33. Randomized double-blind comparison of a calcium antagonist and a diuretic in elderly hypertensives.

National Intervention Cooperative Study in Elderly Hypertensives Study Group. Hypertension 1999;34(5):1129–33.

34. Hansson L, Hedner T, Lund-Johansen P, et al. Randomised trial of effects of calcium antagonists compared with diuretics and β-blockers on cardiovascular morbidity and mortality in hypertension: The Nordic Diltiazem (NORDIL) study. Lancet 2000;356(9227):359–65.

35. Yusuf S, Teo KK, Pogue J, et al. Telmisartan, ramipril, or both in patients at high risk for vascular events. New England Journal of Medicine 2008;358(15):1547–59.

36. MacMahon S, Sharpe N, Gamble G, et al. Randomized, placebo-controlled trial of the angiotensin-converting enzyme inhibitor, ramipril, in patients with coronary or other occlusive arterial disease. Journal of the American College of Cardiology 2000;36(2):438–43.

37. Braunwald E, Domanski MJ, Fowler SE, et al. Angiotensin-converting-enzyme inhibition in stable coronary artery disease. New England Journal of Medicine 2004;351(20).

38. Asselbergs FW, Diercks GFH, Hillege HL, et al. Effects of fosinopril and pravastatin on cardiovascular events in subjects with microalbuminuria. Circulation 2004;110(18):2809–16.

39. Eleuteri E. Effect of amlodipine on the progression of atherosclerosis and the occurrence of clinical events. Italian heart journal Supplement 2001;2(1):85–6.

40. Sacco RL, Diener HC, Yusuf S, et al. Aspirin and extended-release dipyridamole versus clopidogrel for recurrent stroke. New England Journal of Medicine 2008;359(12):1238–51.

41. Randomised trial of a perindopril-based blood-pressure-lowering regimen among 6,105 individuals with previous stroke or transient ischaemic attack. Lancet 2001;358(9287):1033–41.

42. Ogihara T, Nakao K, Fukui T, et al. Effects of candesartan compared with amlodipine in hypertensive patients with high cardiovascular risks: Candesartan antihypertensive survival evaluation in Japan trial. Hypertension 2008;51(2):393–8.

43. Wright JT, Williamson JD, Whelton PK, et al. A randomized trial of intensive versus standard blood-pressure control. New England Journal of Medicine 2015;373(22):2103–16.

44. Hansson L, Lindholm LH, Ekbom T, et al. Randomised trial of old and new antihypertensive drugs in elderly patients: Cardiovascular mortality and morbidity the Swedish trial in old patients with hypertension-2 study. Lancet 1999;354(9192):1751–6.

45. Staessen J, Fagard R, Thijs L, et al. Randomised double-blind comparison of placebo and active treatment for older patients with isolated systolic hypertension. The Systolic Hypertension in Europe (Syst-Eur) Trial Investigators. Lancet. 1997;350(9080):757–64.

46. Telmisartan T, Assessment R. Effects of the angiotensin-receptor blocker telmisartan on cardiovascular events in high-risk patients intolerant to angiotensin-converting enzyme inhibitors: a randomised controlled trial. The Lancet 2008;372(9644):1174–83.

47. Tight blood pressure control and risk of macrovascular and microvascular complications in type 2 diabetes: UKPDS 38. UK Prospective Diabetes Study Group. BMJ (Clinical research ed) 1998;317(7160):703–13.

48. Ogihara T, Saruta T, Rakugi H, et al. Target blood pressure for treatment of isolated systolic hypertension in the elderly: Valsartan in elderly isolated systolic hypertension study. Hypertension 2010;56(2):196–202.

49. Julius S, Kjeldsen SE, Weber M, et al. Outcomes in hypertensive patients at high cardiovascular risk treated with regimens based on valsartan or amlodipine: The VALUE randomised trial. Lancet 2004;363(9426):2022–31.

50. Zanchetti A, Agabiti Rosei E, Dal Palù C, Leonetti G, Magnani B, Pessina A. The Verapamil in Hypertension and Atherosclerosis Study (VHAS): Results of long-term randomized treatment with either verapamil or chlorthalidone on carotid intima-media thickness. Journal of Hypertension 1998;16(11):1667–76.

51. Langford HG, Stamier J, Wassertheil-Smoller S, Prineas RJ. All-cause mortality in the hypertension

detection and follow-up program: Findings for the whole cohort and for persons with less severe hypertension, with and without other traits related to risk of mortality. Progress in Cardiovascular Diseases 1986;29(3 SUPPL. 1):29–54.
